# Bearing characteristics and settlement calculation method of pile groups in beaded karst areas

**DOI:** 10.1371/journal.pone.0337971

**Published:** 2025-12-09

**Authors:** Jie Li, Jiaxing Tao, Juncheng He, Jianhui He, Mingquan Huang, Yan Zhao, Mingjie Zhou

**Affiliations:** 1 China Railway 16th Bureau Group Railway Transportation Engineering Co., Ltd., Guangzhou, Guangdong, China; 2 Key Laboratory of Civil Engineering Diagnosis, Reconstruction and Disaster Resistance of Hebei Province, Zhangjiakou, Hebei, China; 3 School of Mechanics and Civil Engineering, China University of Mining and Technology (Beijing), Beijing, China; China University of Mining and Technology, CHINA

## Abstract

In order to explore the influence of the different position of karst on the bearing capacity of pile group foundation, a theoretical analysis was carried out from the perspective of the reduction rate of pile group effect. Combined with the calculation method of pile foundation settlement, the calculation formula for pile group foundation settlement corresponding to different relative position conditions of karst caves was derived. Finite element numerical simulations were conducted using data from real pile foundation projects, and the results were verified with field measurements. The results demonstrate that the proposed method for analyzing pile bearing characteristics is widely applicable. When the karst cave is located beneath the central area of the pile foundation, the ultimate bearing capacity is relatively high. However, when the karst cave is located in the peripheral area, the ultimate bearing capacity decreases. The conclusions provide a theoretical basis and practical guidance for managing and controlling similar engineering problems.

## 1. Introduction

In recent years, the number of construction projects in karst areas has increased. Studies have pointed out that under the complex geological conditions in karst areas, the stability of foundation projects is particularly important [1–3]. In order to improve the stability of buildings, pile foundations are usually used in karst areas. Pile foundations can stably transfer the upper load to the bearing layer at the pile end or the surrounding rock and soil layer. However, the existence of karst caves changes the bearing characteristics of pile foundations. Therefore, scholars [4–6] have conducted relevant research on this engineering problem.

At present, most pile foundations in karst areas are single pile foundations, and there are many uncertainties in their bearing characteristics under complex geological conditions. Zou et al. [7] discussed the settlement deformation characteristics and stability of bridge pile foundations in beaded karst areas and proposed a load transfer calculation method for the vertical bearing capacity of the pile body with the pile top settlement as the control threshold. Based on the Hoek-Brown failure criterion, Gharsallaoui et al. [8] discussed the hole expansion theory and derived a calculation method for the ultimate bearing capacity of pile ends in karst areas. These analyses are primarily based on theoretical formulas derived for idealized cave and pile configurations and do not adequately account for the complex geology of karst areas. This includes the variability and irregular distribution of caves and their influence on piles. The study focused on a single cave type and did not provide a systematic analysis of variations in geometry, location, or scale. As a result, the conclusions are subject to certain limitations. The influence of caves on pile foundations extends beyond load transfer and is manifested through cave characteristics. Features such as their position [9] and height [10] significantly affect the bearing capacity of the piles. Li et al. [11] investigated the effects of pile diameter, the ratio of top plate thickness to pile diameter, and the ratio of cave width to pile diameter on the stability of karst foundations, and proposed a three-dimensional method for effectively evaluating their bearing capacity. These studies indicate that the bearing behavior of piles in karst areas is influenced by multiple factors, including stratum conditions, cave geometry, and pile parameters. The theoretical framework concerning single piles in karst regions has been relatively well established. With the expansion of civil engineering projects, pile groups are increasingly employed in foundation construction. However, as a complex system, the bearing capacity of a pile group foundation exhibits significant differences in analysis and calculation compared with a single pile. Consequently, researchers have increasingly focused on the bearing characteristics of pile group foundations.

Current research on pile group foundations primarily investigates factors affecting bearing capacity, such as the pile group effect [12], pile spacing [13], pile diameter [14], and pile length [15]. The pile group effect is an important factor affecting the bearing capacity of pile group foundations. It refers to the mutual influence of the mechanical behavior between pile foundations in a pile group foundation, which causes the bearing capacity and settlement of each pile in the pile group system to be different from that of a single pile. Therefore, many scholars have carried out a lot of systematic research work on the influence of pile group effect on the bearing capacity of pile foundation. Relying on indoor effective continuous model tests, Takewaki et al. [16] analyzed the impact of pile group effect on buildings. The research results pointed out that the existence of pile group effect can effectively reduce the inter-story displacement of buildings and enhance the pile end resistance in soft foundations. Tang et al. [17] reported that increasing pile spacing and length, while reducing pile diameter, can mitigate the pile group effect. Kim et al. [18] conducted indoor sand model tests, which showed that, under identical loads, pile groups settled over twice the amount observed in single piles, and pile spacing had a significant influence on the pile group effect. Liu et al. [19] analyzed the influence of pile-soil relative stiffness on pile group effect and verified it by finite element numerical simulation. The results show that the influence of pile group effect increases with the increase of pile-soil relative stiffness. Under special geological conditions, the bearing characteristics of pile group foundation are more complicated. Ye et al. [20] analyzed the deformation mechanism and internal force response law of high-rise pile group foundation (HRPG) on slope foundation. The research results indicate that slope, pile cap height, and lateral load have significant effects on the displacement and bending moment of group pile foundations. The displacement and bending moment increase with the increase of slope, but decrease with the increase of pile cap. However, the aforementioned studies did not examine in detail how the pile group effect influences the bearing capacity of foundations under varying pile lengths, number of piles, and diameters. Accordingly, Al-Omari et al. [21] investigated load transfer in saturated and unsaturated soils through aluminium alloy model piles arranged in various group configurations. They found that ultimate load and bearing capacity increase with the number of piles and as soil conditions change from saturated to unsaturated. Fattah et al. [22] conducted 94 dynamic load tests on single piles and pile groups in dry soils with relative densities of 30% and 50%, and with pile spacings of three, four, and five times the pile diameter. They measured acceleration and displacement, and found that the acceleration amplitude increased with both frequency and soil density. In addition, Fattah et al. [23]measured vertical and horizontal displacements under dynamic loads. The results of the study found that higher vibration frequencies reduce the oscillation of waves. Pile spacing is an important factor affecting the displacement of pile top. Moreover, the internal force decreases slightly with the increase of pile spacing. Fattah, et al. [24] studied the load sharing mechanism of pile foundations under different soil conditions, focusing on the distribution ratio of bearing force and tip bearing capacity. The results show that for saturated soil conditions, when a single pile reaches the ultimate bearing capacity, the loads borne by the bearing and tip are roughly equal. For unsaturated soil, the load is mainly borne by the tip. In addition, the proportion of load transferred to the pile shaft increases with the increase in the number of piles in the pile group. However, the aforementioned studies have primarily focused on the overall settlement performance of pile group foundations, and less attention has been given to individual piles at different positions within the group. Wang et al. [25] conducted finite element simulations of large-scale, long pile groups and found that outer piles showed bearing behavior similar to single piles, whereas inner piles differed significantly in their bearing behavior. Noman et al. [26] analyzed the effects of varying pile spacings on load transfer and bearing capacity in pile groups under vertical loads in collapsible loess, and examined the behavior of corner, side, and center piles. Fattah, et al. [27] explored the contribution of factors such as installation method, relative density, soil plug removal, pile aspect ratio, friction and end resistance to the ultimate bearing capacity of pile foundations. The results showed that the contribution of the middle pile of an asymmetric pile group foundation is 1.3 times the load borne by the edge piles. The above studies examined variations in pile group bearing characteristics at different pile positions but have largely neglected their mechanical behavior under non-uniform loads, including inclined and vertically eccentric loads. Therefore, Zhou et al. [28] analyzed the effects of inclined loads on the bearing capacity of pile groups. They employed p-y curves to calculate the bearing characteristics of pile groups under horizontal loads and the pile-soil interaction under vertical loads, and determined the load-displacement and bending moment distributions of the pile foundation via an iterative method. Based on the limit analysis theorem, Di Laora et al. [29] examined the influence of vertical eccentric loads and proposed a formula for bearing capacity. Souri et al. [30] employed the finite element method to study the effects of pile spacing and pile group configuration on static lateral resistance. The damaged, hybrid, and vertical structures exhibited the highest, intermediate, and lowest efficiency, respectively. An increase in spacing significantly reduced the axial reaction force. The *p* multipliers varied notably among the structures, with the greatest effect observed in the damaged structure. Most previous studies have focused on pile group behavior in non-karst areas. With the growing use of pile groups in karst regions, the combined complexity arising from pile groups and karst caves requires further investigation of the evolution of their mechanical behavior.

Pile group foundations are widely used in civil engineering projects in karst areas due to their adaptability, which highlights the importance of investigating both bearing capacity and stability. Lei et al. [31] investigated the bearing mechanism and failure mode of the rock strata at the ends of the piles of the underlying karst cave bridge group, and proposed a calculation method for the ultimate bearing capacity of the relevant pile foundation by combining the limit analysis method. Niu et al. [32] established a stability analysis model of the underlying karst cave under the pile group foundation based on the finite element numerical method and discussed the influence of the pile group on the stability of the cave. The study focused on stability and bearing characteristics with underlying caves, without considering the influence of through caves on bearing behavior. Therefore, taking the Qihe Bridge as a case study, Ou et al. [33] examined the bearing characteristics of pile foundations and the impact of caves when multiple piles in a group intersect them, and further elucidated the mechanisms by which caves influence pile foundation behavior. At present, there are relatively few studies on pile group foundations in karst areas, and most of the research contents are usually about the “whole” pile group passing through a large cave. However, in actual projects, the size of the cave may not be large enough to be penetrated by the entire pile group foundation. Therefore, the influence of the relative position of the underlying cave on the bearing capacity of the pile group foundation cannot be ignored. Based on the above analysis, this paper intends to explore the influence of the relative position of the cave on the bearing characteristics of the pile group foundation from the perspective of the reduction rate of the pile group effect. Through theoretical analysis, on-site monitoring and numerical simulation, the evolution characteristics of the mechanical behavior of the pile group foundation under complex karst geological conditions are systematically studied. Finally, relying on the pile foundation project of the Science and Technology Manufacturing Park project in Bai-Yun District, Guangzhou City, Guangdong Province, the reliability of the theoretical model is verified, and the potential influence of the cave position on the overall stability and bearing performance of the pile group foundation is analyzed.

## 2. Calculation of settlement and bearing capacity of group pile foundation in karst area

### 2.1. Calculation of reduction rate of pile foundation at each position in pile group system

**Fig 1:**
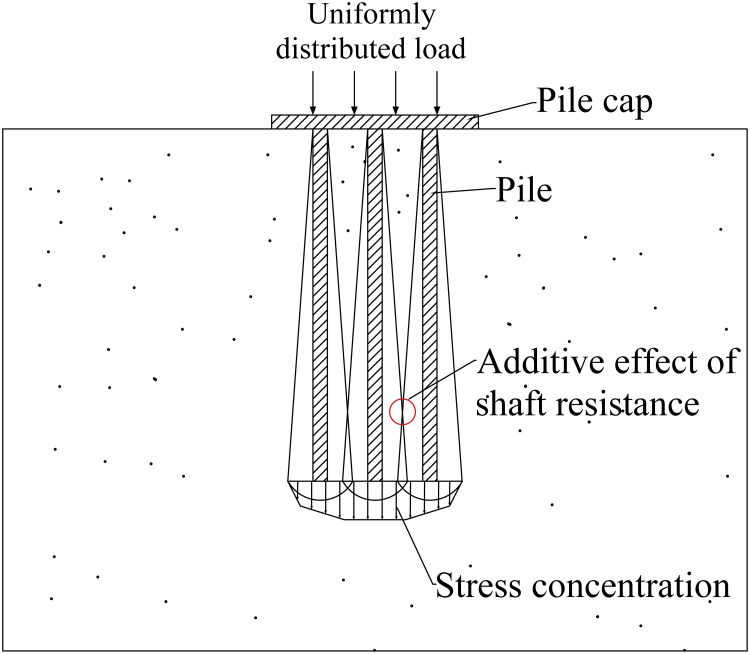
Pile group effect of pile group foundation.


As shown in Fig 1, due to the existence of the pile group effect, the bearing characteristics of the pile group foundation are somewhat different from those of the single pile foundation. Studies have shown that the bearing capacity of the pile group foundation is not a simple addition of the bearing capacity of each single pile, but requires consideration of the mutual influence between each pile foundation. Related research [19,34,35] describes the above problem by introducing the concept of pile group effect coefficient.

$$W_{U}=\eta aP_{U}$$ (1)

where *W*_*U*_ represents the ultimate bearing capacity of pile groups, *P*_*U*_ is the ultimate bearing capacity of single pile, *a* is the number of piles in the pile group foundation, and *η* is the pile group effect coefficient.

At present, there are five main methods for calculating the pile group effect coefficient: the solid perimeter method, the Converse-Labarre method [36], the Seiler-Keeney method [37], the partial coefficient method, and the stress superposition method [38]. Among them, the stress superposition method comprehensively considers the influence of multiple factors such as pile spacing, number of piles, pile length, and surrounding soil characteristics, and can better reflect the pile group effect of pile group foundations in karst areas. The core idea of the stress superposition method is to regard the pile group system as multiple independent pile foundations, analyze the stress field induced by each pile foundation under the load, and then superimpose the stress fields generated by each pile to obtain the overall stress distribution of the pile group system.

Based on the concept of stress superposition, we make the following assumptions about the theoretical derivation part. The soil is assumed to be uniformly distributed throughout the entire area. The vertical load on the pile foundation is assumed to be uniformly distributed, with any uneven distribution at the pile top neglected. The interaction between pile foundations is characterised by contact stiffness and transmitted forces, while complex nonlinear interactions are neglected. The soil is assumed to be sufficiently deep, such that boundary effects on the pile group can be neglected.

The pile group effect coefficient for a rectangular pile group with an m × n configuration is defined in the stress superposition method as follows:


$$\eta =\frac{\text{1}}{1+\lambda }$$ (2)

$$\lambda =2A_{1}\frac{m-1}{m}+2A_{2}\frac{n-1}{n}+4A_{3}\frac{\left(m-1\right)\left(n-1\right)}{mn}$$ (3)

where λ represents the average reduction factor accounting for stress superposition within the pile group. The parameter *m* represents the number of transverse piles, while *n* represents the number of longitudinal piles. The reduction rate represents the decrease in the bearing capacity of a single pile due to the influence of surrounding piles within the group. *A*₁ represents the reduction rate resulting from overlapping stresses of adjacent transverse piles. *A*₂ represents the reduction rate resulting from overlapping stresses of adjacent longitudinal piles. *A*₃ represents the reduction rate resulting from overlapping stresses of diagonally adjacent piles. For a single pile or a portion of the pile group, the average reduction coefficient fails to represent the influence of the pile group effect. Accordingly, the average reduction factor formula is reformulated to provide an expression representing the constraint imposed by the pile group on each pile, resulting in the reduction rate for a single pile under the effect of neighbouring piles.


$$A_{1}=\frac{\text{1}}{3r_{1}}-\frac{\text{1}}{2L\tan\varphi }$$ (4)

$$A_{2}=\frac{\text{1}}{3r_{2}}-\frac{\text{1}}{2L\tan\varphi }$$ (5)

$$A_{3}=\frac{\text{1}}{3\sqrt{r_{1}^{2}+r_{2}^{2}}}-\frac{\text{1}}{2L\tan\varphi }$$ (6)

where *φ* is the weighted average of the internal friction angles of each soil layer within the pile body, m is the number of transverse piles, *n* is the number of longitudinal piles, *L* is the pile length, *r*_1_ is the transverse pile spacing, and *r*_2_ is the longitudinal pile spacing. Based on engineering design and field measurements, these parameters are determined from the pile foundation layout.

**Fig 2:**
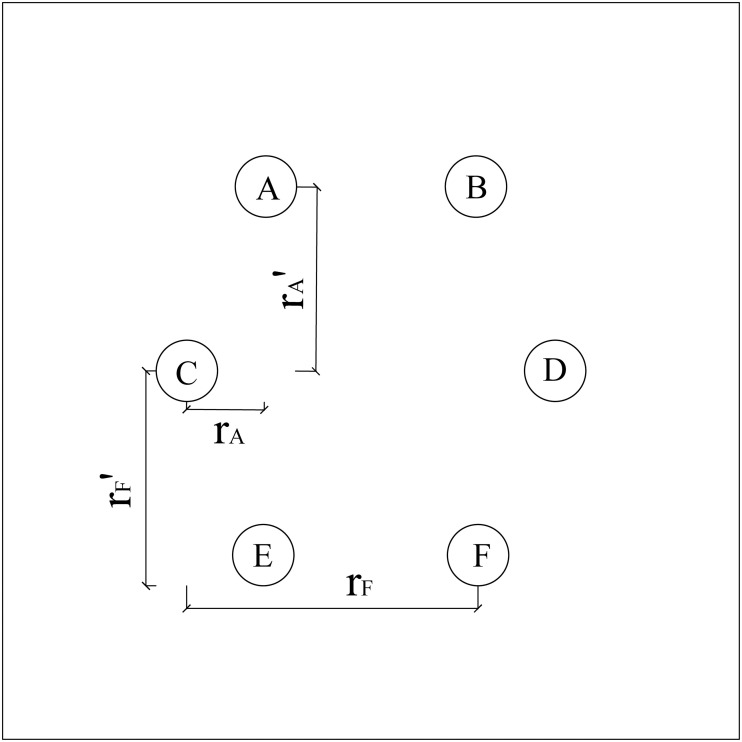
Non-rectangular arrangement of pile foundations.


As shown in Fig 2, non-rectangular pile group foundations vary from standard rectangular pile groups with respect to their layout. For example, the reduction rate of pile C depends on the surrounding piles A, B, D, E, and F, and is calculated as follows:

$$A_{A}=\frac{\text{1}}{3\sqrt{r_{A}^{2}+r_{A}^{{}^{\prime }2}}}-\frac{\text{1}}{2L\cdot \tan\varphi }$$ (7)

$$A_{F}=\frac{\text{1}}{3\sqrt{r_{F}^{2}+r_{F}^{{}^{\prime }2}}}-\frac{\text{1}}{2L\cdot \tan\varphi }$$ (8)

Similarly, the reduction effects of piles A, B, D, E, and F on pile C depend on their respective distances. The total reduction rate for pile C is therefore given by the sum: *A*_*A*_ + *A*_*B*_ + *A*_*D*_ + *A*_*E*_ + *A*_*F*_.

### 2.2. Calculation of bearing capacity of single pile foundation in pile group system

The reduction rate is an important indicator to measure the degree of reduction in vertical bearing capacity caused by the interaction between piles in a pile group system. It reflects the weakening effect of the stress generated by the diffusion of the friction resistance on the side of the adjacent piles on the bearing capacity of a single pile. Based on the above analysis, the actual bearing capacity of a single pile in a pile group system can be expressed as follows:


$$P_{U}^{\prime }=P_{U}\left(1-A\right)$$ (9)

where *P’ U* represents the actual bearing capacity of a single pile in the pile group system, and *A* is the sum of the reduction rates of the single pile.

In a pile group foundation, the actual bearing capacity of the piles at the corner, edge and center positions is different due to the different degrees of influence of overlapping stresses on the three positions.

**Fig 3:**
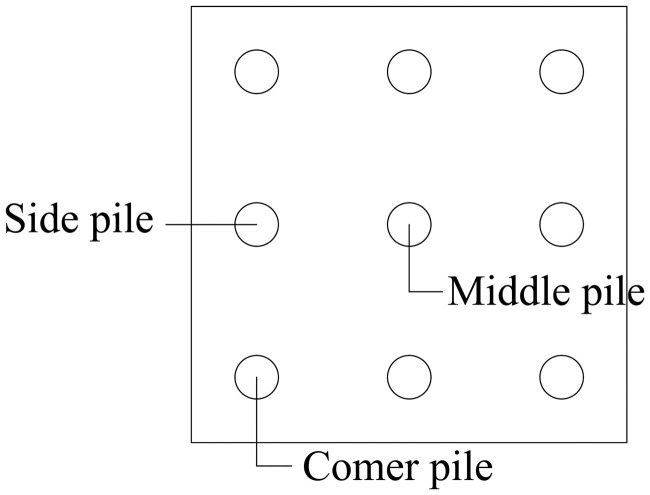
3 × 3 pile group foundation.

For pile group foundations, increasing the pile spacing can effectively reduce the impact of the pile group effect [39] and improve the bearing capacity of the pile group foundation. According to engineering needs and economic factors, the design of the pile spacing of the pile group foundation cannot be too large, resulting in a waste of space and economy, nor can it be too small, resulting in too high a pile group effect of the pile group foundation and reducing the overall bearing capacity. Research [40] has shown that when the pile spacing of a pile group foundation is greater than 6 times the pile diameter, the influence of the pile group effect on the pile foundation can be ignored. The optimal pile spacing for pile group foundation is usually 3–6 times the pile diameter. For large pile groups, a single pile in the pile group will only be affected by the adjacent piles and will not be affected by the piles farther away. That is, a pile will be affected by at most 8 piles surrounding it. In smaller groups, such as the typical 2 × 2 configuration, the limited number of piles does not allow clear differentiation between corner, side, and middle piles. In contrast, a 3 × 3 pile group provides a clear representation of these positions. Accordingly, a 3 × 3 pile group foundation is adopted in this study. As shown in Fig 3, the middle, side, and corner piles are influenced by eight, five, and three adjacent piles, respectively.

Based on the above analysis, it can be seen that the reduction rate of corner piles is *A*_1_ + *A*_2_ + *A*_3_, the reduction rate of side piles is 2 *A*_1_ + *A*_2_ + 2 *A*_3_ = *A*_1_ + 2 *A*_2_ + 2 *A*_3_, and the reduction rate of center piles is 2 *A*_1_ + 2 *A*_2_ + 4 *A*_3_.

In conventional analyses of pile-soil interaction, the linear superposition approach is often employed, where different factors are assumed to act independently and their effects are evaluated separately before being combined through direct addition. However, pile-soil interaction in engineering practice shows nonlinear characteristics, particularly when subjected to high loads. The soil stress-strain response is highly nonlinear, with yielding, plastic deformation and heterogeneous behavior, which cannot be accurately captured by linear models. Additionally, stress diffusion and interactions among piles in a pile group lead to variations in the bearing capacity loss of each pile depending on the loading conditions of adjacent piles. Consequently, linear superposition methods are prone to overestimating such losses. To address this, a hyperbolic model is introduced to provide a more accurate representation of pile-soil side friction, thus reducing the overestimation inherent in linear superposition methods.

For friction pile foundation or end-bearing friction pile, the bearing capacity of the pile foundation is mainly borne by the side friction resistance of the pile [13], which can generally be calculated by the following formula:


$$Q_{S}=\int_{0}^{L}\tau_{i}\left(z\right)S\left(z\right)dz$$ (10)

where *Q*_S_ represents the total pile side friction. *τ*_*i*_(*z*) is the pile side friction at depth z of the *i*-th soil layer, *S*_*i*_(*z*) is the pile side surface area of the i-th soil layer. *z*_*i*−1_ and *z*_*i*_ are the upper and lower limit depths of the i-th soil layer, respectively.


$$\tau_{i}\left(z\right)=\frac{\tau_{i0}}{1+\beta_{i}\cdot Z}$$ (11)

$$\tau_{i0}=c_{i}+\sigma_{i}\cdot \tan(\phi_{i})$$ (12)

where *τ*_*i*0_ represents the initial frictional resistance of the *i*-th soil layer, *c*_*i*_ denotes the cohesion, *σ*_*i*_ the effective normal stress, and *ϕ*_*i*_ the internal friction angle of the layer. Neglecting groundwater effects, the effective normal stress in the *i*-th soil layer is expressed as *σ*_*i*=_*γz*, where *γ* is the soil unit weight and *z* is the depth of the layer. The friction decay coefficient *β*_*i*_ can be obtained from experimental results or engineering experience.

**Fig 4:**
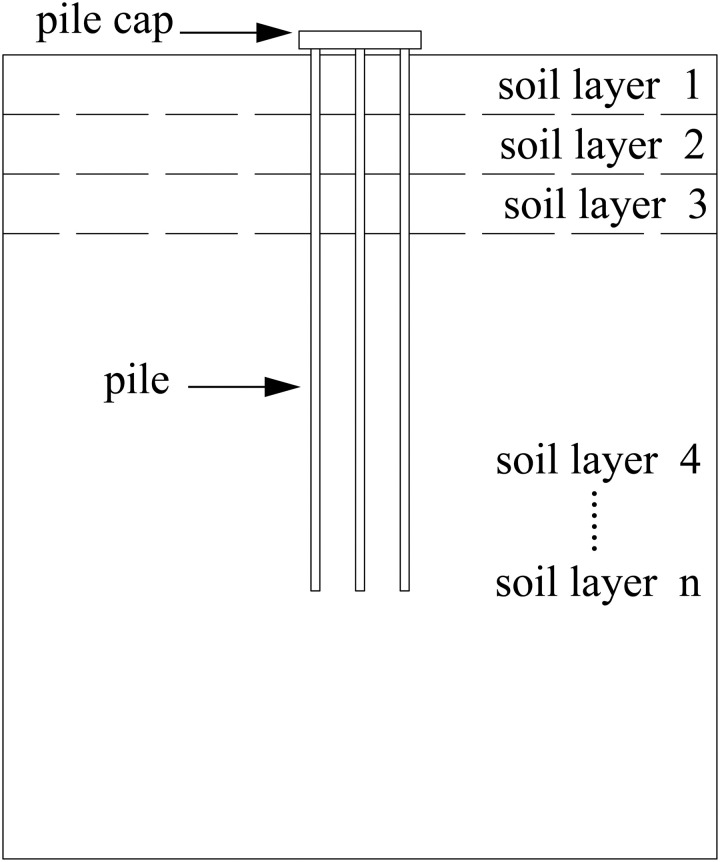
Schematic diagram of multi-layer soil.

As shown in Fig 4, for multi-layer soil, when solving the pile side friction, it is necessary to solve each layer of soil and then superimpose the calculation.

The actual pile side friction of a single pile in a pile group foundation can be expressed as:


$$Q_{S}^{\prime }=Q_{S}\left(1-A\right)$$ (13)

Combined with the calculation method of the reduction rate of pile groups, it can be obtained that the actual bearing capacity of corner piles is *Q^’^_S_*_1_ = *Q^’^_S_*·[1-(*A*_1_ + *A*_2_ + *A*_3_)], the actual bearing capacity of side piles is *Q^’^_S2_* =*Q^’^_S_*·[1-(2*A*_1_+2*A*_2_ + *A*_3_)], and the actual bearing capacity of center piles is *Q^’^_S3_* = *Q^’^_S_*·[1-(2*A*_1_+2*A*_2_ + 4*A*_3_)].

### 2.3. Actual bearing capacity of each pile foundation when the cave is located at different positions in the pile foundation

A large number of practical results have confirmed [19,41] that when there is an underlying cave beneath the pile foundation or the pile foundation itself passes through the cave, the cave will have a certain impact on the bearing capacity of the pile foundation. Especially for through-type friction pile foundations in karst areas, the existence of caves will greatly affect the bearing capacity of the pile foundation. The “voids” formed by the caves make the contact between the soil and the pile foundation fail, resulting in the loss of pile side friction resistance here. The lateral friction of a single pile passing through a cave can be expressed as:


$$Q_{S}^{{}^{\prime \prime }}=\int_{0}^{L}\tau \left(z\right)S\left(z\right)dz-\sum {\mathrm{\backslash nolimits}}_{i=1}^{x}\int_{L_{1}}^{L_{2}}\tau \left(z\right)S\left(z\right)dz$$ (14)

where *x* is the number of caves, *L*1 and *L*_2_ represent the locations of the upper and lower boundaries of the cave respectively.

For pile group foundations, due to the existence of pile group effect, the calculation of pile side friction resistance when the pile foundation passes through a cave is different from that when an ordinary single pile passes through a cave. For example, when a corner pile passes through a cave, the actual pile side friction can be expressed as: *Q” S1* = *Q” S*[1-(*A*_1_ + *A*_2_ + *A*_3_)]. Similarly, the actual pile side friction resistance when the side pile or middle pile passes through the cave can be expressed as follows, *Q” S2* = *Q” S*[1-(2*A*_1_+2*A*_2_ + *A*_3_)], *Q” S3* = *Q” S*[1-(2*A*_1_+2*A*_2_ + 4*A*_3_)].

From the above analysis, it can be seen that due to the influence of the pile group effect, when the pile foundations at different positions in the pile group system pass through the underlying cave, the pile side friction loss is different. When the cave is located in the central area of the pile group system, the bearing capacity loss is the smallest. The loss of pile side friction in a friction pile group foundation directly affects the overall bearing characteristics of the pile foundation.

### 2.4. Calculation of pile group foundation settlement when karst caves are located at different positions

The settlement calculation of pile group foundations in soft soil can usually be obtained by the layer-wise summation method [35,42], namely:


$$N=\sum {\mathrm{\backslash nolimits}}_{i=1}^{b}\frac{\sigma_{Z}}{E_{i}}△z_{i}+\frac{Q_{j}}{L}\varepsilon_{e}\frac{1}{E_{C}A_{C}}$$ (15)

where *E*_*i*_ is the elastic modulus of the *i*-th soil layer, *b* is the number of soil layers, ∆*z*_*i*_ is the thickness of the *i*-th rock and soil layer, *Q*_*j*_ is the load borne by the pile group foundation, *E*_*C*_ is the elastic modulus of the pile body, *A*_*C*_ is the total cross-sectional area of the pile, *ε*_*e*_ is the compression coefficient of the pile body. Its value is related to the type of pile and the pile diameter ratio *L*/*D* For friction piles, the following empirical formula is generally used to determine it:


$$\varepsilon_{e}=\{\;\;\;\;\begin{array}{cc} {}*20c\frac{\text{2}}{\text{3}}\mathrm{\backslash vspace}1mm & \frac{L}{D}\leq 30\\ -\frac{\text{1}}{120}\cdot \frac{L}{D}+\frac{11}{12} & \mathrm{\backslash vspace}1mm30<\frac{L}{D}<50\mathrm{\backslash vspace}1mm\\ \frac{\text{1}}{\text{2}} & \frac{L}{D}\geq 50 \end{array}$$ (16)

*σ*_*Z*_ represents the additiona*l* vertical stress caused by the pile side friction:


$$\sigma_{Z}=\frac{Q_{S\cdot total}}{9L}I_{S}$$ (17)

*I*_*S*_ is the influence coefficient related to the pile side friction resistance, which can be obtained by the following equation.


$$I_{S}=\frac{1-v_{i}}{E_{i}}$$ (18)

Combining equations (14)–(18), the pile group settlement is:


$$N=\sum {\mathrm{\backslash nolimits}}_{i=1}^{b}\frac{Q_{S\cdot total}I_{S}}{9E_{i}L}\Delta z_{i}+\frac{Q_{j}}{L}\varepsilon_{e}\frac{\text{1}}{E_{C}A_{C}}$$ (19)

When piles at different positions in the pile group foundation pass through the cave, the corresponding settlement of the pile group system is different. When the corner pile passes through the cave, the total bearing capacity of the pile group can be expressed as *Q” S*1 + 3*Q’ S*1 + 4*Q’ S*2 + *Q’ S*3, and the settlement of the pile group is:


$$N=\sum {\mathrm{\backslash nolimits}}_{i=1}^{b}\frac{\left(Q_{S1}^{{}^{\prime \prime }}+3Q_{S1}^{\prime }+4Q_{S2}^{\prime }+Q_{S3}^{\prime }\right)I_{S}}{9E_{i}L}\Delta z_{i}+\frac{Q_{j}}{L}\varepsilon_{e}\frac{\text{1}}{E_{C}A_{C}}$$ (20)

Similarly, when the side piles or middle piles pass through the cave, the pile foundation settlement can be expressed as follows:


$$N=\sum {\mathrm{\backslash nolimits}}_{i=1}^{b}\frac{\left(4Q_{S1}^{\prime }+Q_{S2}^{{}^{\prime \prime }}+3Q_{S2}^{\prime }+Q_{S3}^{\prime }\right)I_{S}}{9E_{i}L}\Delta z_{i}+\frac{Q_{j}}{L}\varepsilon_{e}\frac{\text{1}}{E_{C}A_{C}}$$ (21)

$$N=\sum {\mathrm{\backslash nolimits}}_{i=1}^{b}\frac{\left(4Q_{S1}^{\prime }+4Q_{S2}^{\prime }+Q_{S3}^{{}^{\prime \prime }}\right)I_{S}}{9E_{i}L}\Delta z_{i}+\frac{Q_{j}}{L}\varepsilon_{e}\frac{\text{1}}{E_{C}A_{C}}$$ (22)

**Fig 5:**
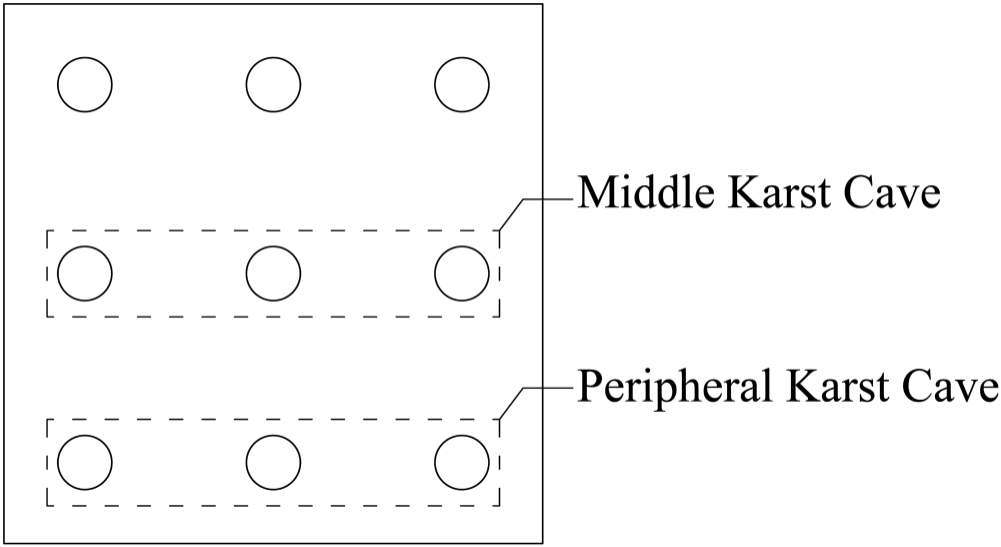
Multiple pile foundations passing through a cave.

As shown in Fig 5, for a pile group system with multiple piles passing through a karst cave, when calculating the pile foundation settlement, the pile side friction resistance of the pile foundation part passing through the karst cave needs to be “subtracted”. At this time, the pile side friction resistance can be calculated by formula (14). That is, when calculating the total pile side friction resistance of the pile group, the pile foundation part passing through the karst cave needs to be “calculated separately”.

Assuming that three piles in the middle area of the pile group system pass through the cave, the settlement of the pile group foundation can be obtained by the following formula:


$$N=\sum {\mathrm{\backslash nolimits}}_{i=1}^{b}\frac{\left(4Q_{S1}^{\prime }+2Q_{S2}^{{}^{\prime \prime }}2Q_{S2}^{\prime }+Q_{S3}^{{}^{\prime \prime }}\right)I_{S}}{9E_{i}L}\Delta z_{i}+\frac{Q_{j}}{L}\varepsilon_{e}\frac{\text{1}}{E_{C}A_{C}}$$ (23)

When three piles at the edge of the pile group foundation pass through the cave, the settlement of the pile group foundation is:


$$N=\sum {\mathrm{\backslash nolimits}}_{i=1}^{b}\frac{\left(2Q_{S1}^{\prime }+2Q_{S1}^{{}^{\prime \prime }}+3Q_{S2}^{\prime }+Q_{S2}^{{}^{\prime \prime }}+Q_{S3}^{\prime }\right)I_{S}}{9E_{i}L}\Delta z_{i}+\frac{Q_{j}}{L}\varepsilon_{e}\frac{\text{1}}{E_{C}A_{C}}$$ (24)

## 3. Project overview and on-site monitoring

### 3.1. Project overview

**Fig 6:**
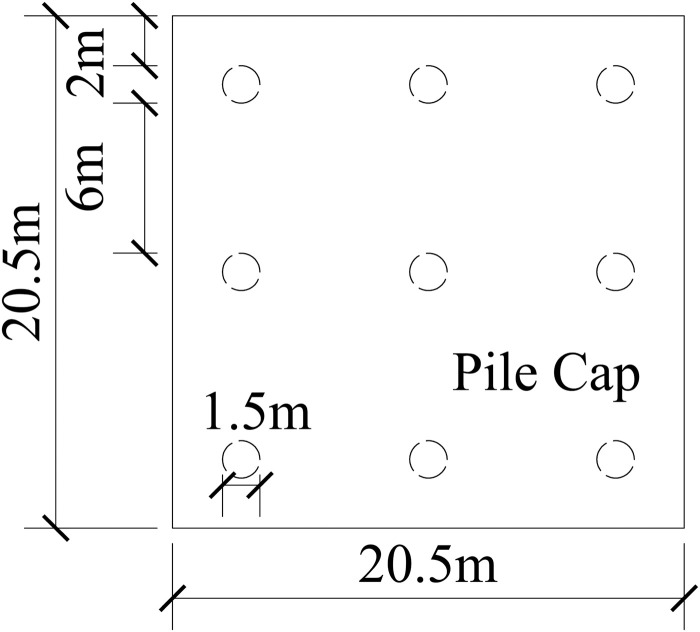
Plan layout of pile group foundation.

This paper takes the pile foundation project of the Science and Technology Manufacturing Park project in Bai-Yun District, Guangzhou City, Guangdong Province as the background. The karst development of the site belongs to a typical covered karst cave. The surface rock and soil layers are mixed fill, silty soil, silty clay, and limestone from top to bottom. Among them, the thickness of the mixed fill is 5 ~ 15 m, the thickness of the silty soil is 4 ~ 20 m, the thickness of the silty clay is 10 ~ 30 m, and the thickness of the limestone is 50 ~ 90 m. The physical parameters of the soil layer are shown in Table 1. The pile foundation mainly adopts C35 reinforced concrete bored piles with a length of 91 m and a diameter of 1.5 m, and the pile spacing is 6 m. The foundation is made of C35 concrete with dimensions of 20.5 × 20.5 × 3 m. The plan layout is shown in Fig 6.

**Table 1:** Mechanical parameters of rock and soil.

	Density/kg·m^-3^	Elastic modulus/MPa	Cohesion/MPa	Poisson’s ratio	Internal friction angle/°
Miscellaneous fill soil	1650	3	0.01	0.3	10
Silty soil	1720	3.5	0.015	0.33	12
Silty clay	1930	10	0.07	0.35	20
Limestone	2200	265	0.5	0.25	35

**Fig 7:**
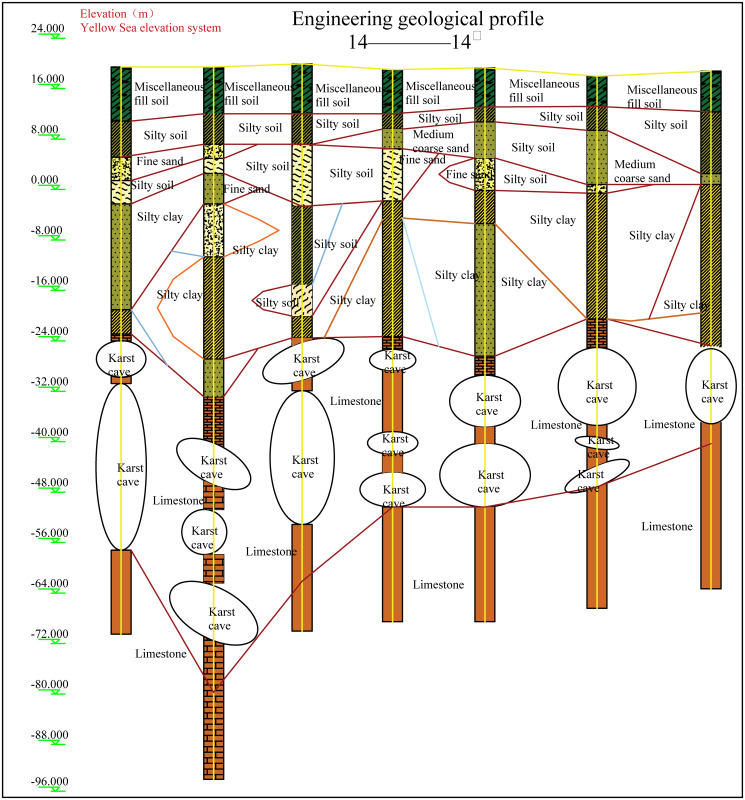
Geological profile.

The geological survey results show that 59 of the 68 boreholes revealed underlying karst caves. According to the standard “General Code for Building and Municipal Foundations” (GB 55003−2021), the hole rate of the boreholes is 86.76%, and the karst rate of the borehole line is 21.33%, which indicates a site with strong karst development. The caves are distributed in a beaded pattern, a distinctive karst landform consisting of a series of stone pillars, caves and gullies that resemble beads strung together, giving rise to the term “beaded” caves. These caves typically develop through geological processes such as water erosion and karstification, appearing as either continuous or scattered, fairly regular stone pillars or karst voids. Beaded caves can be categorised according to their degree of filling, which may be filled, partially filled or unfilled; their size, ranging from small to large; and their depth, from shallow caves of tens of metres to deep caves exceeding hundreds of metres. As shown in Fig 7, the actual caves display a shallow beaded pattern. Geological exploration indicates a low cave filling rate, which has little effect on the cave properties.

**Fig 8:**
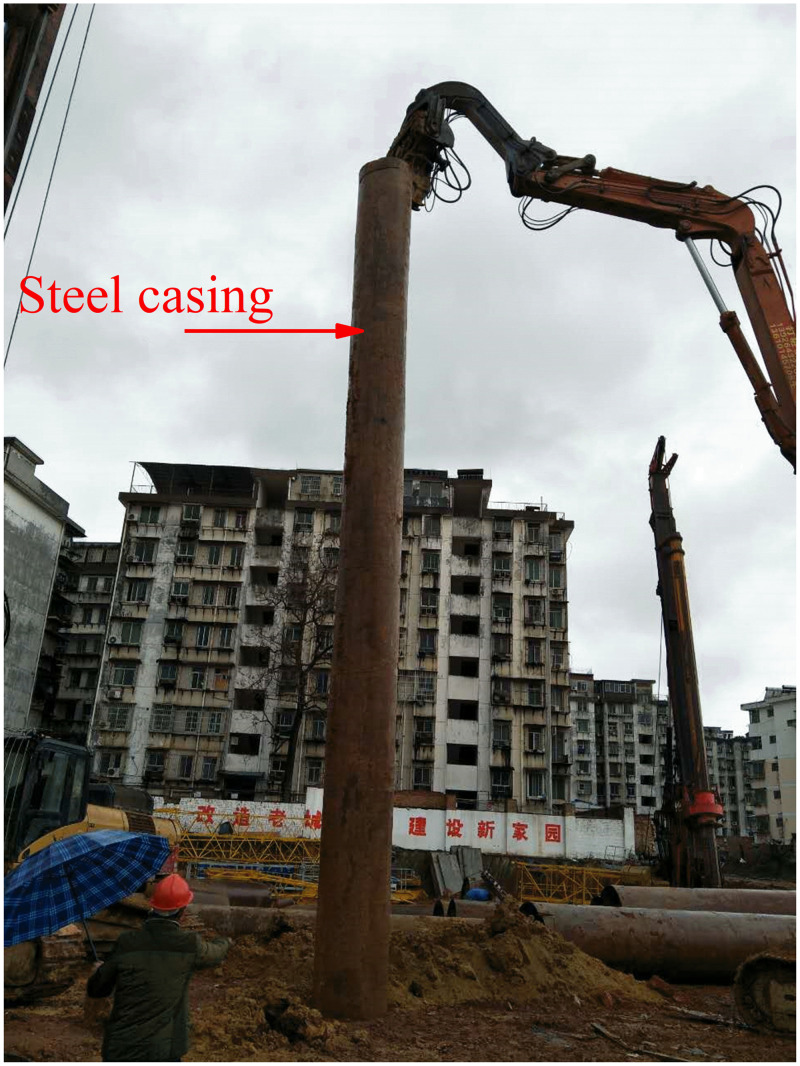
Steel casing application.

As shown in Fig 8, for large-volume caves, the location of the cave is accurately located before pile foundation construction, and steel casing is used for follow-up protection measures.

### 3.2. Analysis of rock and soil micromorphology and pore structure characteristics

**Fig 9:**
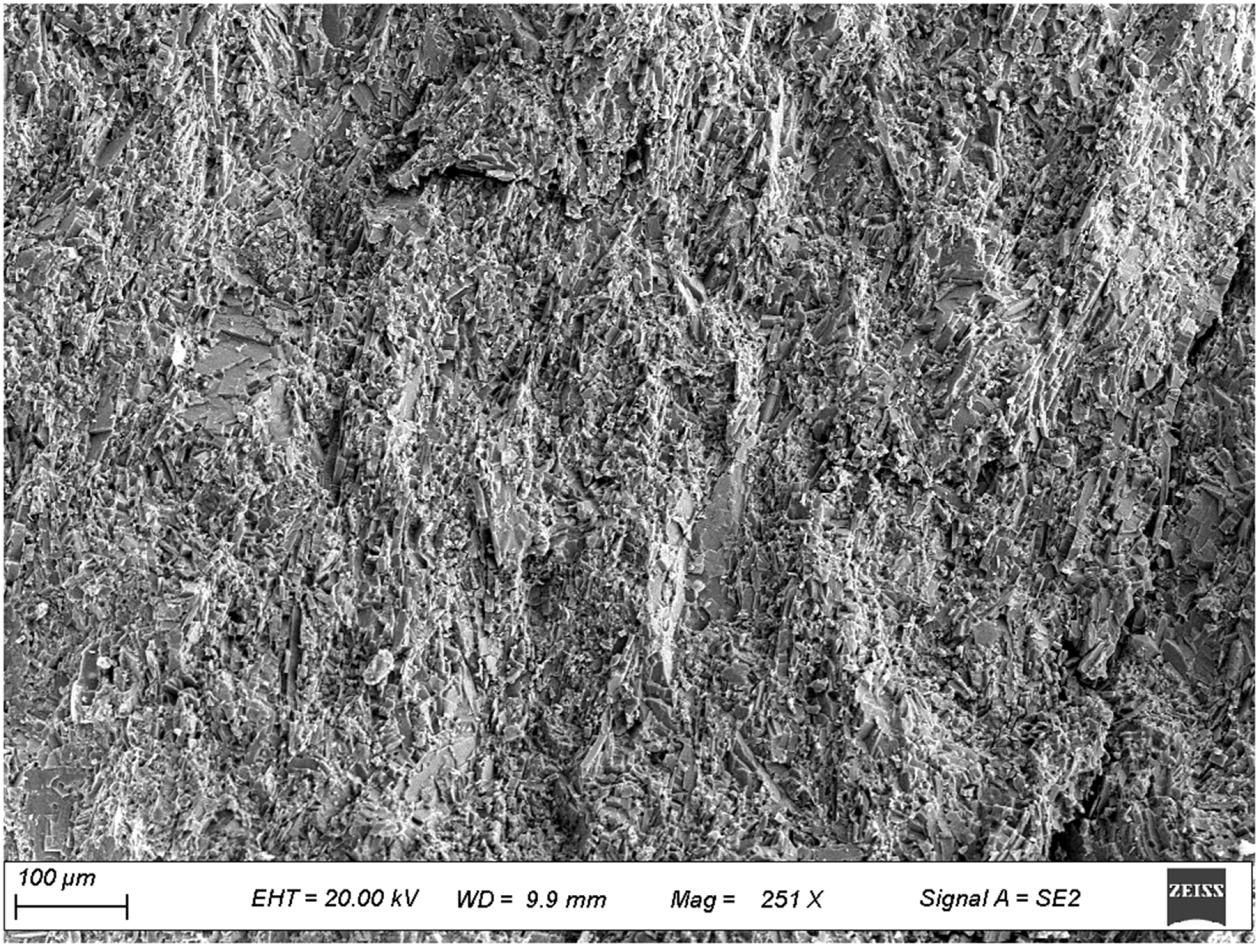
Microscopic structure of limestone away from the karst cave.

**Fig 10:**
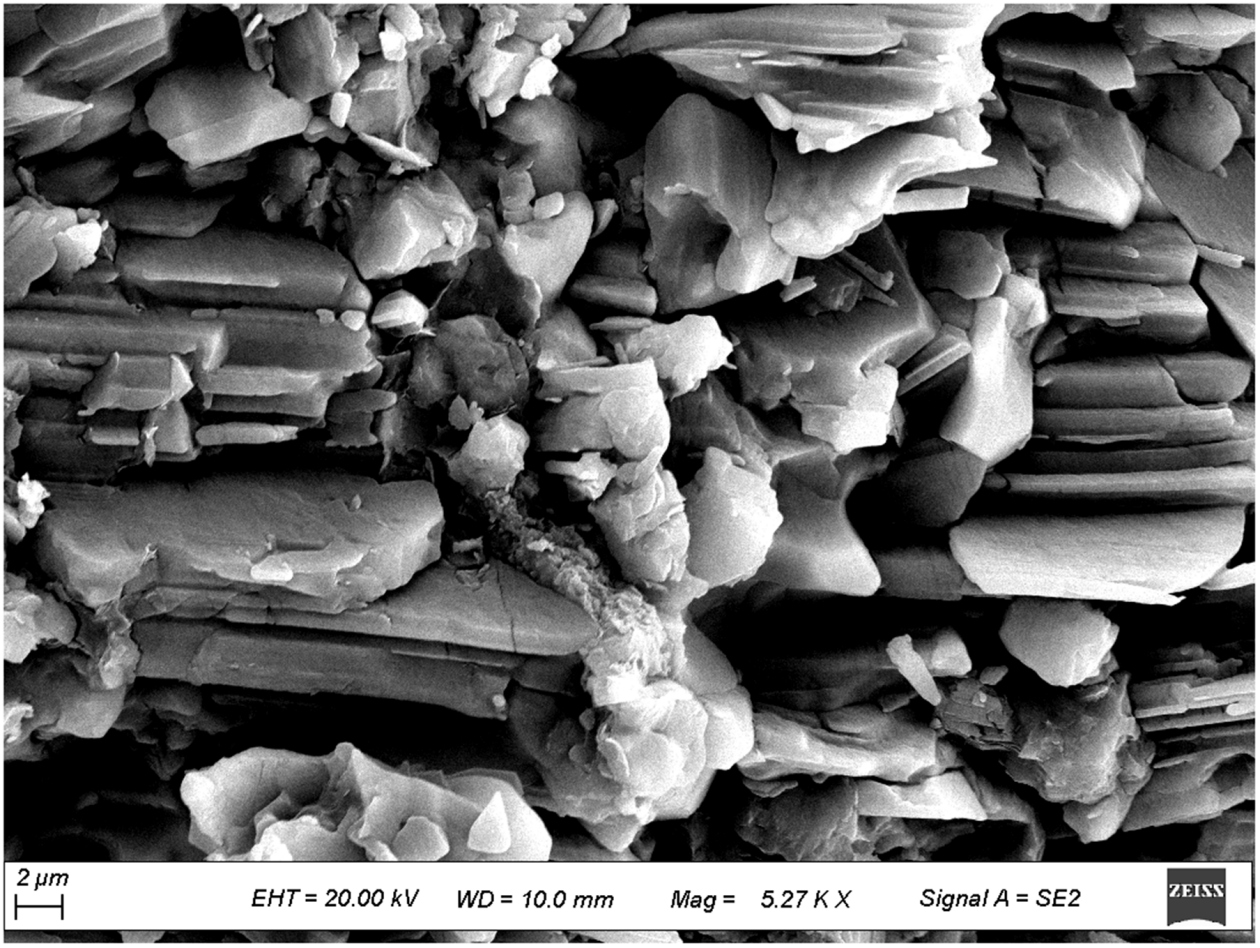
Microscopic structure of limestone near the karst cave.

**Fig 11:**
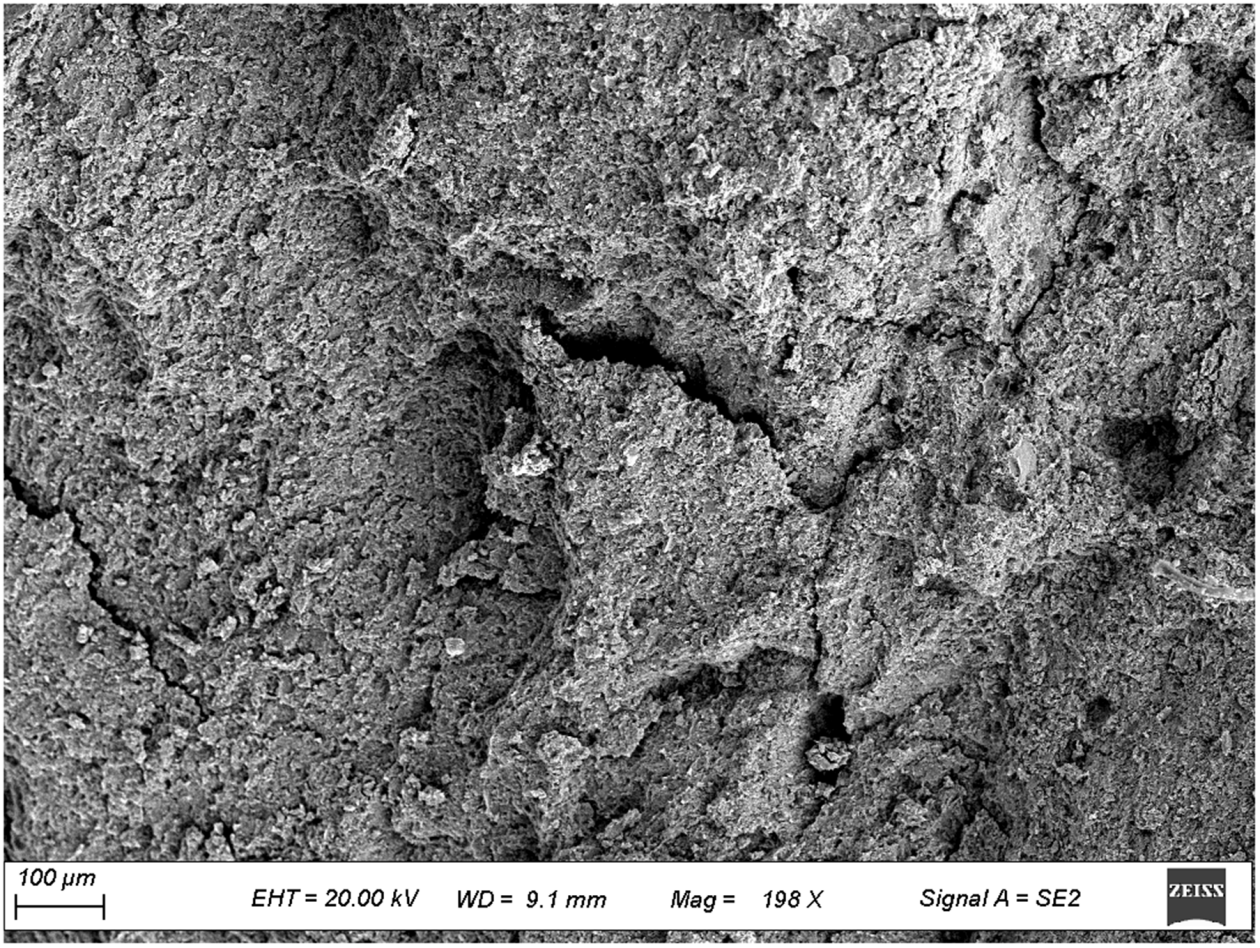
Microscopic structure of silty clay away from the karst cave.

**Fig 12:**
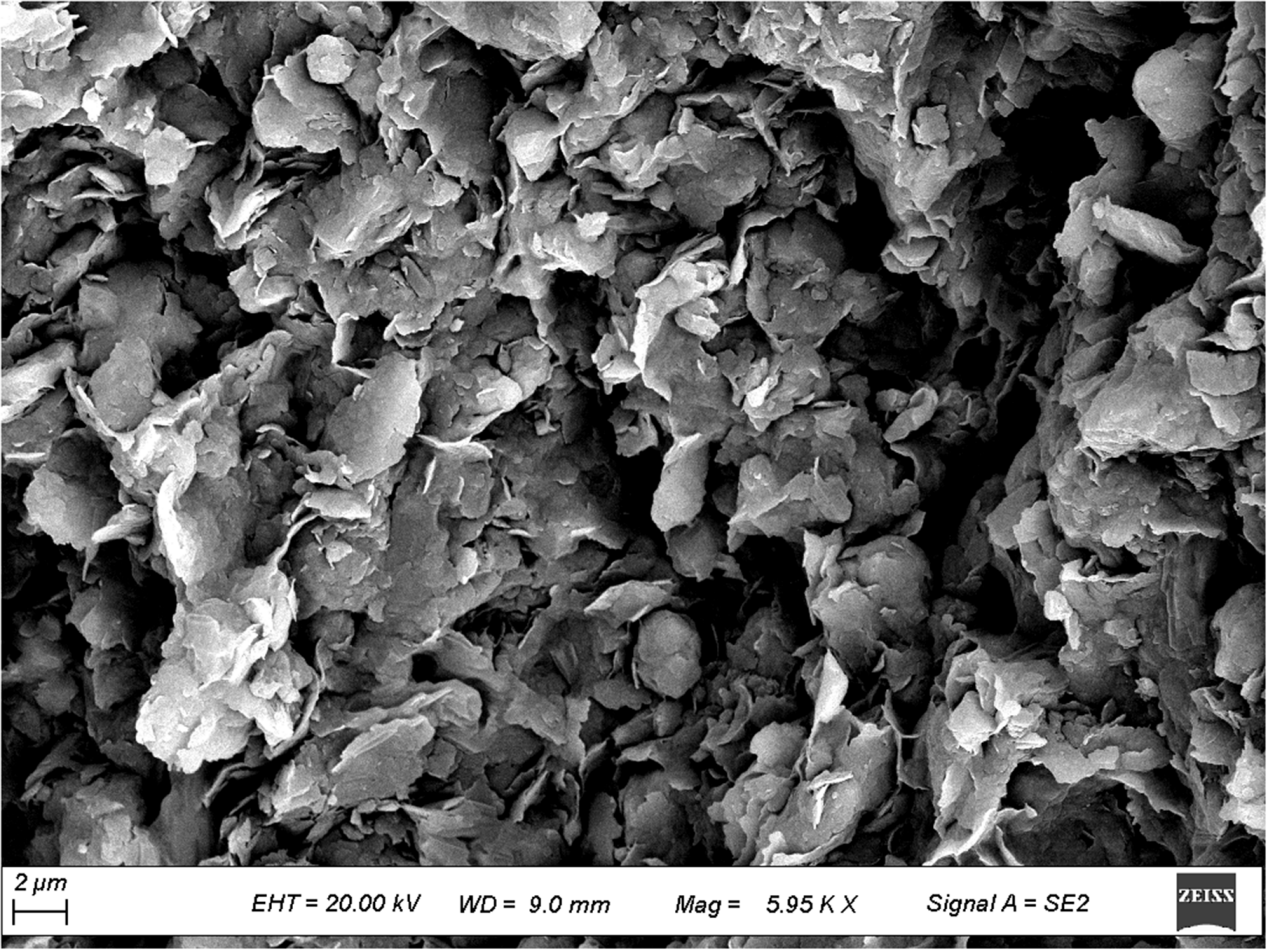
Microscopic structure of silty clay near the karst cave.

To further investigate the micromechanical properties of the pile-rock-soil interface in the karst area, scanning electron microscopy (SEM) observations were performed on typical rock and soil samples collected from the site. Fig 9–12 presents microstructural images of limestone and silty clay sampled at different distances from the cave. Figs 9 and 10 show the SEM results for limestone, while Figs 11 and 12 display those for silty clay. Images 9 and 11 correspond to samples from locations distant from the cave, representing the undisturbed microstructure, whereas images 10 and 12 depict the microstructure near the cave, which exhibits enhanced erosion effects.

#### 3.2.1. Analysis of microstructural characteristics of limestone samples.

As shown in Fig 9, the limestone located away from the cave exhibits dense particles and strong structural continuity. Cementing minerals are visible locally, and the particles are clearly interlocked. This dense structure enhances the shear strength of the pile-rock interface and provides stable frictional resistance under vertical loads. Furthermore, the low pore connectivity limits interfacial slip during deformation, indicating good mechanical stability of the foundation. As shown in Fig 10, the limestone adjacent to the cave contains well-developed pores, distinct particle boundaries, loose cementation, significant local weathering and erosion, and evident structural degradation. Meanwhile, limestone samples were taken from areas closer to the karst caves, where the erosion effect from karst groundwater was more pronounced, weakening the cementation of rock particles and increasing porosity near the caves. Researches [43] show that hydraulic action can disrupt the cemented structure of rock masses, and increase the risk of structural instability. Under stress, this rock mass is prone to local shear slip, reducing the shear bearing capacity of the pile-rock interface. In engineering practice, friction pile foundations are commonly employed for such rock conditions.

#### 3.2.2. Analysis of microstructural characteristics of silty clay samples.

As shown in Fig 11, silty clay located away from the cave area has loosely arranged particles with weak inter-particle contacts, high porosity, and irregular interface structures, indicating low cementation and limited shear strength. Under engineering conditions, it cannot provide continuous and stable pile-soil side friction. As shown in Fig 12, silty clay adjacent to the cave exhibits a looser microstructure, indistinct particle boundaries, and local microcracks and fractures. These features suggest that combined cave hydraulic erosion and stress disturbance have further weakened soil cementation and exacerbated structural degradation. The compromised structure is prone to micro-slip and shear failure during loading, significantly reducing frictional resistance at the pile-soil interface and increasing the risk of settlement. Its bearing stability, particularly under prolonged loading, therefore requires careful assessment.

### 3.3. Pile group foundation settlement monitoring

After completion of the pile foundation project, on-site tests were conducted under controlled conditions to evaluate the actual bearing capacity and performance characteristics of the pile group foundation. To ensure the accuracy of monitoring, all measurements were carried out under standardised conditions. The test area was fenced, and benchmarks were calibrated to minimise external interference. The test pile foundations were distributed as AZK06, AZK09, AZK12, AZK13, and AZK16, and the design load was 400 kPa. The soil types at the measurement points were consistent with the findings from the site investigation. The overlying soil layer comprises miscellaneous fill, silty clay, and silty soil, underlain by highly weathered limestone bedrock. All three edge piles of the AZK06 pile group foundation pass through large, beaded caves. In the AZK09 pile group foundation, piles at both the edges and the centre all have caves, most of which are scattered and unevenly distributed. All three central piles of the AZK12 pile group foundation pass through large, beaded caves. The middle pile of the AZK13 pile group foundation passes through a beaded cave, with no caves elsewhere. In the AZK16 pile group foundation, the three piles at the upper edge and in the lower right corner all have caves, most of which are small, scattered, and beaded. AZK06 and AZK12, which have similar soil environments but different cave locations, were used for comparison to observe the differences in bearing capacity caused by cave location.

**Fig 13:**
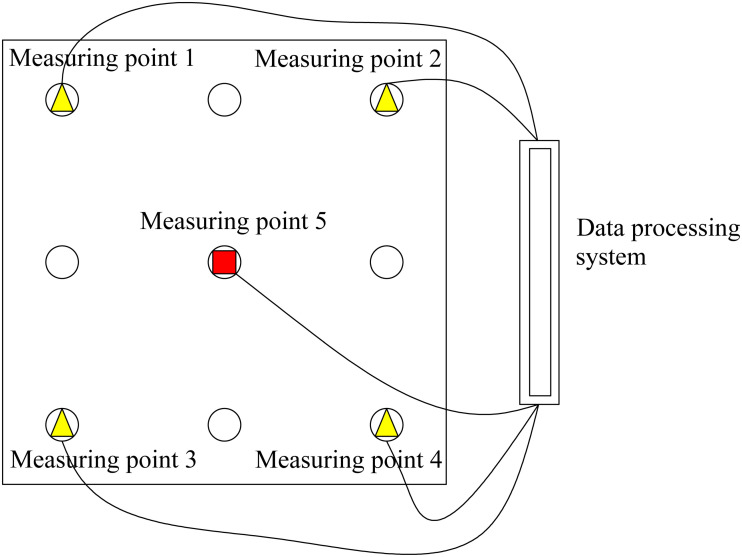
Schematic diagram of settlement on-site monitoring.

As shown in Fig 13, settlement observation points are arranged at the four corner piles and the middle pile on the surface of the pile group foundation. The observation points of the four corner piles are used to monitor whether the pile group foundation exhibits uneven settlement. The observation point of the middle pile is used to monitor the overall settlement of the foundation. A high-precision displacement sensor optoNCDT2300−100 is connected to the data acquisition system for remote real-time monitoring. In order to ensure that the sensor can perform high-precision measurements, high-precision instruments such as total stations are used to determine the monitoring points during the installation process. Then, the sensor mounting bracket is fixed to the customized metal base, which is firmly connected to the pile foundation concrete through chemical anchor bolts. At the same time, high-strength bolts and shock-absorbing rubber pads are used to double-fix the sensor to avoid measurement errors caused by external factors such as construction vibration and mechanical disturbance. After installation, the sensor is calibrated to correct the zero point and linear deviation of the sensor. The sensor signal transmission cable adopts double-layer shielded cable. For the strong electromagnetic interference source in the site, a metal shielding net is installed to reduce the impact on the signal transmission effect. Two optoNCDT2300−100 sensors are deployed at each monitoring point for redundant measurement. By comparing the measurement data of the two sensors, outliers can be eliminated and data reliability can be improved.

The loading method for pile group settlement monitoring adopts the heap loading method, which uses high-density concrete blocks for loading and lifts them by crane to evenly distribute them on the cap, simulating the uniformly distributed load on the cap. Each pile load is 20% of the design load. After the load was applied, it was maintained for 30 minutes to allow the pile foundation settlement to stabilize. Loading is stopped when the design load is reached or the settlement rate is monitored to change suddenly or increase too fast. The settlement size and settlement rate of each loading level are counted. If the settlement growth rate does not change significantly after the load reaches the design load, we will continue to load with small loads, with each level of load being 10%. When the load rate increases sharply, we need to stop loading immediately, observe whether the settlement is stable, and record the load size at this time. The monitoring results are shown in Tables 2–6. After data processing, it is found that the ultimate bearing capacity of AZK06 pile group foundation is 440 kPa, the ultimate bearing capacity of AZK09 pile group foundation is 440 kPa, the ultimate bearing capacity of AZK12 pile group foundation is 480 kPa, the ultimate bearing capacity of AZK13 pile group foundation is 520 kPa, and the ultimate bearing capacity of AZK16 pile group foundation is 480 kPa.

**Table 2:** Monitoring results of AZK06 pile group foundation (mm).

Load/kPa	Measuring point 1	Measuring point 2	Measuring point 3	Measuring point 4	Measuring point 5
80	9.27	9.36	10.56	11.19	10.15
160	18.65	18.15	20.76	20.49	19.64
240	26.76	26.03	27.53	28.77	27.64
320	34.86	34.04	35.94	36.21	35.73
400	43.47	43.09	45.36	44.41	44.29
440	53.37	53.76	58.64	55.72	55.12
480	63.89	63.32	64.63	65.03	64.24

**Table 3:** Monitoring results of AZK09 pile group foundation (mm).

Load/kPa	Measuring point 1	Measuring point 2	Measuring point 3	Measuring point 4	Measuring point 5
80	9.01	8.89	11.79	10.86	9.98
160	17.39	17.08	19.67	19.28	18.23
240	25.56	25.90	27.89	27.65	26.02
320	32.96	33.61	36.62	36.43	34.65
400	42.27	42.75	45.73	45.49	43.27
440	46.14	46.53	50.54	51.13	47.92
480	49.89	50.32	54.63	55.03	52.46

**Table 4:** Monitoring results of AZK12 pile group foundation (mm).

Load/kPa	Measuring point 1	Measuring point 2	Measuring point 3	Measuring point 4	Measuring point 5
80	4.32	4.57	5.22	4.49	4.81
160	13.25	13.13	13.85	13.04	12.97
240	20.24	19.98	20.73	20.13	20.36
320	27.43	27.23	27.94	27.21	27.47
400	37.06	36.95	37.64	37.26	37.35
440	42.19	42.26	42.71	42.19	42.39
480	45.36	45.31	45.95	45.24	45.27

**Table 5:** Monitoring results of AZK13 pile group foundation (mm).

Load/kPa	Measuring point 1	Measuring point 2	Measuring point 3	Measuring point 4	Measuring point 5
80	5.27	5.45	5.64	5.93	5.48
160	11.43	11.74	11.82	11.41	12.09
240	18.74	18.87	18.26	18.20	18.52
320	25.23	25.10	25.38	26.01	25.85
400	33.15	33.45	33.74	33.49	34.38
480	41.07	40.75	40.43	40.24	41.51
520	46.48	47.19	47.34	47.84	47.95
560	56.41	56.75	57.24	56.84	57.61

**Table 6:** Monitoring results of AZK16 pile group foundation (mm).

Load/kPa	Measuring point 1	Measuring point 2	Measuring point 3	Measuring point 4	Measuring point 5
80	6.32	6.57	6.22	6.49	6.81
160	13.25	13.13	13.85	13.04	12.97
240	20.24	19.98	20.73	20.13	20.36
320	27.43	27.23	27.94	27.21	27.47
400	37.06	36.95	37.64	37.26	37.35
440	42.19	42.26	42.71	42.19	42.39
480	45.36	45.31	45.95	45.24	45.27
520	57.23	57.06	57.91	57.26	57.58

## 4. Numerical simulation analysis of bearing characteristics of pile group foundation in beaded strata

### 4.1. Establishment and verification of finite element model

**Fig 14:**
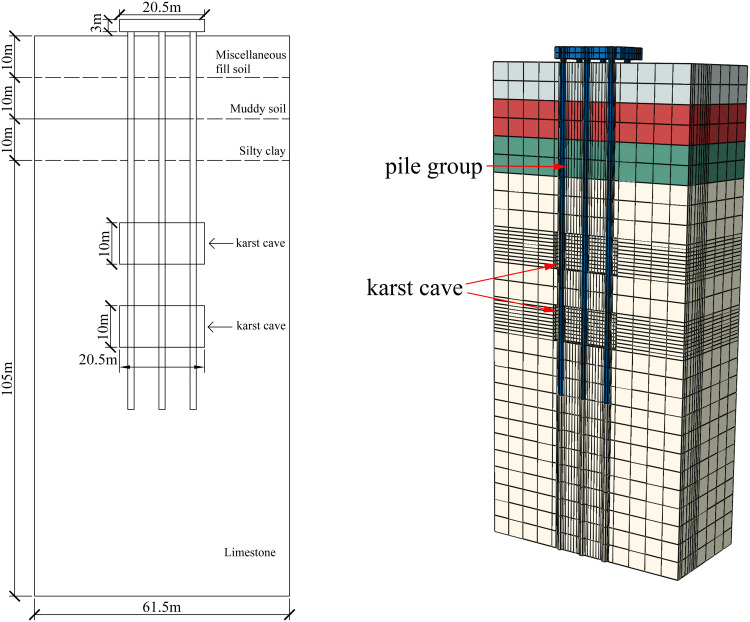
Finite element numerical model and specific dimensions.

A calculation model for the pile foundations at construction sites AZK06 and AZK12 was established through the ABAQUS finite element software. During the calculation process, the numerical model restored the size of the pile group foundation in the actual project at a 1:1 ratio. The location of the cave is consistent with the actual measurement, located at a depth of 45–55 m and 65–75 m in the soil. Assuming that the beaded caves are of the same size and are all regular rectangular parallelepipeds, the cave dimensions are 20.5 × 3.75 × 10 m. To eliminate the influence of boundary conditions, the height of the soil model is 1.5 times the part of the pile foundation in the soil, that is, 135 m. The length and width are 3 times the side length of the pile group foundation cap, that is, 61.5 m. The model employs eight-node solid elements to accurately represent the interaction between the pile foundation and the surrounding rock-soil mass. A locally refined mesh is applied to the soil near the caves to enhance the accuracy and reliability of the simulation results. The specific model establishment is shown in Fig 14.

In order to obtain more accurate results of numerical simulation, it is necessary to add boundary conditions to model. Normal constraints are applied to the sides of the model to allow the soil to move vertically. The bottom surface is fully constrained, and the top surface of the model is a free surface without constraints. For the contact part between pile and soil, the pile is set as the main contact surface and the soil is set as the slave contact surface. In addition, the tangential behavior at the pile-soil contact surface is simulated using a penalty function, the friction factor is 0.35, and the normal behavior is hard contact. The pile group system consists of two parts: the cap and the pile foundation. The cap and the pile foundation are merged into a whole through Boolean operation and the boundaries are retained.

**Fig 15:**
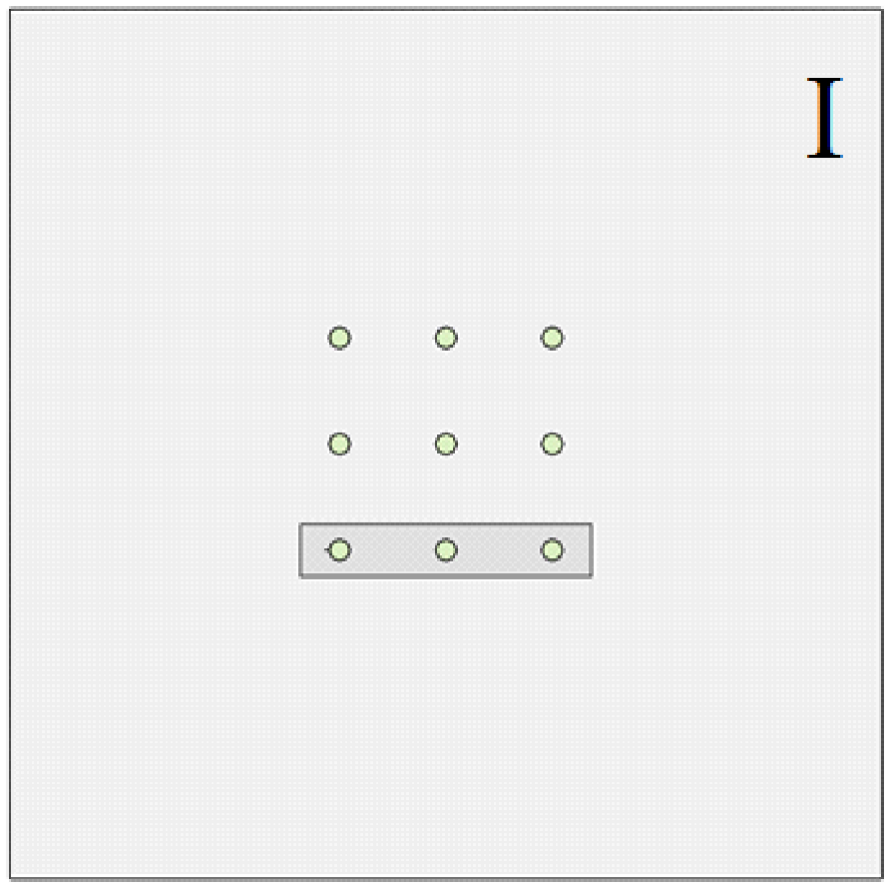
Case I.

**Fig 16:**
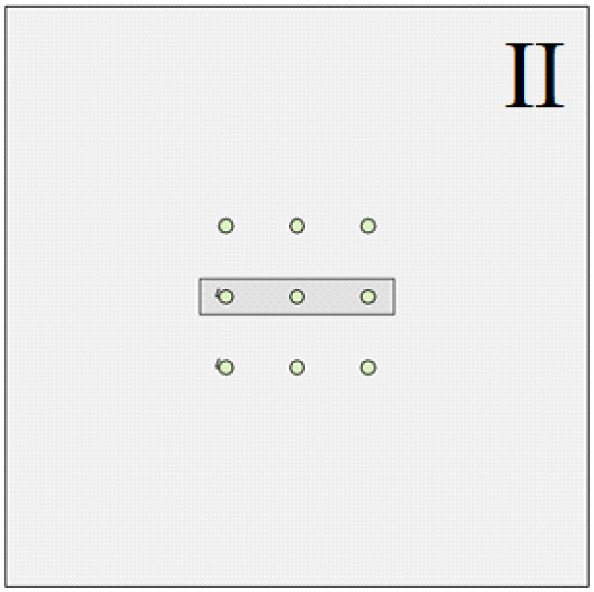
Case II.

**Fig 17:**
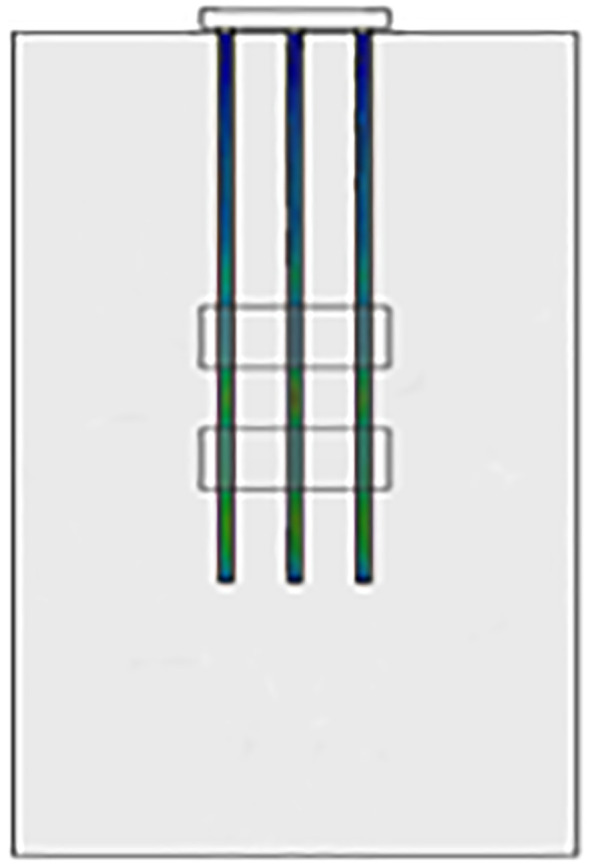
Model cross-section diagram.

The soil layer distribution from top to bottom is as follows: miscellaneous fill 10 m, silty soil 10 m, silty clay 10 m, and limestone 105 m. In order to study the influence of the relative position relationship between the cave and the pile group on the bearing capacity of the pile foundation, relevant numerical simulation comparative experiments were carried out. As shown in Figs 15–17, the changing characteristics of the pile foundation bearing characteristics are analyzed in two special cases: the cave is located at the edge and the middle of the pile foundation area. For the convenience of description, the situation in Fig 15 is regarded as I, and the situation in Fig 16 is regarded as II.

The Mohr-Coulom model is used for the rock and soil around the pile, and the pile body and the cap are simplified into an integrated model, using a linear elastic model. The specific physical and mechanical parameters are shown in Table 7.

**Table 7:** Mechanical parameters of piles and surrounding rock and soil.

	Density/kg·m^-3^	Elastic modulus/MPa	Cohesion/MPa	Poisson’s ratio	Internal friction angle/°
Pile (Cap)	2400	26000	—	0.20	—
Miscellaneous fill	1650	3	0.01	0.3	10
Silt soil	1720	3.5	0.015	0.33	12
Silty clay	1930	10	0.07	0.35	20
Limestone	2200	265	0.5	0.25	35

Uniformly distributed load can better reflect the bearing condition of pile group system. Therefore, this paper adopts the method of applying uniformly distributed load to carry out graded loading. The whole loading process is divided into 6 levels, and the load increases by 100 kPa at each level. The loading surface of the pile group system is defined as the pile cap top face. To achieve uniform load application, this surface is coupled to its centroid, where a concentrated force equivalent to the total applied load is imposed. This approach provides an efficient representation of the uniform load condition within the model. The reliability of the finite element model was verified by comparing numerical simulation with on-site monitoring. The comparison is based on settlement data from the AZK06 and AZK12 pile group foundations, as shown in Tables 8 and 9.

**Table 8:** AZK06 Comparison of pile foundation settlement data (mm).

Load/kPa	80	160	240	320	400	480	520
On-site monitoring	10.15	19.64	27.64	35.73	44.29	55.12	64.24
Numerical simulation	11.95	21.26	29.94	38.76	47.29	58.62	67.52

**Table 9:** AZK12 Comparison of pile foundation settlement data (mm).

Load/kPa	80	160	240	320	400	480	520
On-site monitoring	10.08	18.42	26.17	34.94	43.54	52.95	60.13
Numerical simulation	10.54	19.13	27.39	36.59	45.69	55.53	63.52

As shown in Table 8 and 9, the relative error between the numerical simulation and the field monitoring results ranges from 3% to 5%. However, the comparison results show that the settlement of the pile group foundation is larger because the actual shape of the cave is a beaded circular cave and the soil condition is more complicated.

### 4.2. Analysis of numerical simulation results

**Fig 18:**
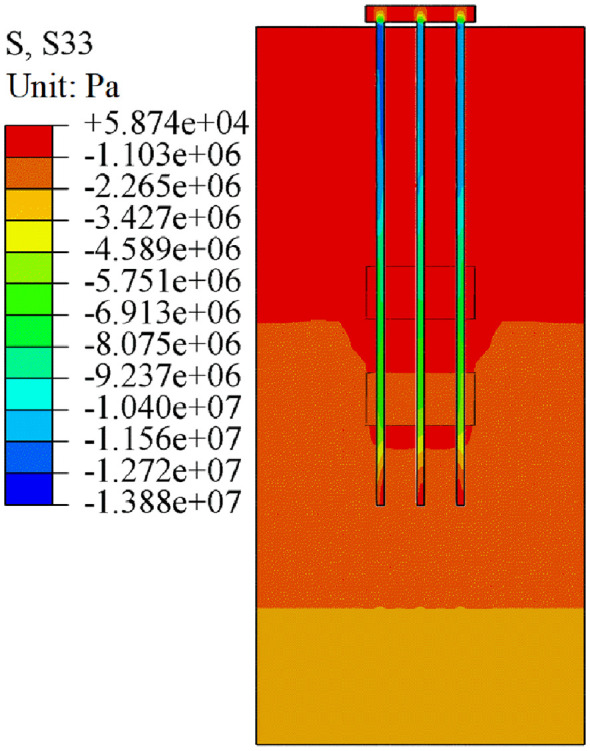
Stress cloud diagram of case I.

**Fig 19:**
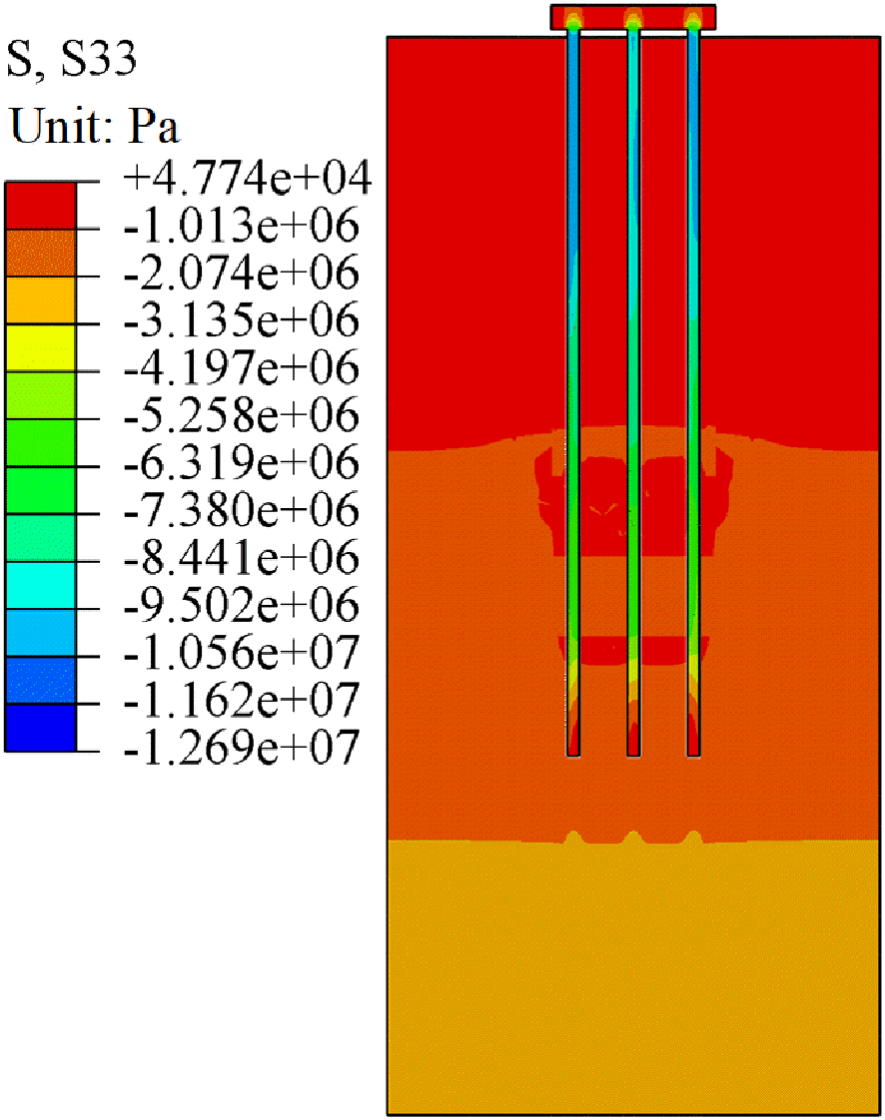
Stress cloud diagram of case II.

The analysis is carried out by taking the uniformly distributed load of 300 kPa on the pile group foundation cap as an example. As shown in Figs 18, 19, the pile-soil stress under both working conditions showed obvious stratification characteristics, and the stress changes of piles and soils at different depths were quite different. For the soil layer, when the pile cap is subjected to uniformly distributed load, the stress concentration of the soil layer near the pile body is obvious. The main concentrated areas are the overlying rock and soil layer, the middle rock layer, and the lower rock layer of the beaded cave. Due to the existence of the karst cavity, the stress of the pile body cannot be evenly transmitted to the interior of the soil layer, resulting in stress concentration in the karst area. At the junction of the pile group foundation and the cave, stress concentration induces shear failure in the surrounding soil. As shown in Fig 18, stress concentration is pronounced near the cave and in the interbedded rock layers, affecting a wide area and resulting in punching-shear failure. During load transfer, a portion of the load is mobilized through pile side friction, leading to a reduced load at the pile tip, mitigated stress concentration, and punching-shear failure. As shown in Fig 19, the load transfer mechanism under case II is similar to that under case I. However, with the cave located at the centre of the pile group foundation, load transfer through the central pile is more constrained, resulting in smaller transferred load and weaker stress concentration compared to case I. For the pile group foundation itself, especially for the three pile foundations that pass through the beaded caves, the existence of the caves causes obvious abnormal distribution of pile body stress. The existence of the cave changes the stress transfer path of the load on the pile body. The side friction of the pile foundation part passing through the cave disappears, and a higher stress concentration is formed in the pile end area. The different locations of the caves lead to different stress changes in the soil and pile foundation in the two cases. When the cave is located at the edge of the pile group, the stress concentration between the pile and soil is more obvious. When the cave is located in the middle area of the pile group, the stress concentration effect is smaller than that in the edge area due to the constraint of other pile foundations.

**Fig 20:**
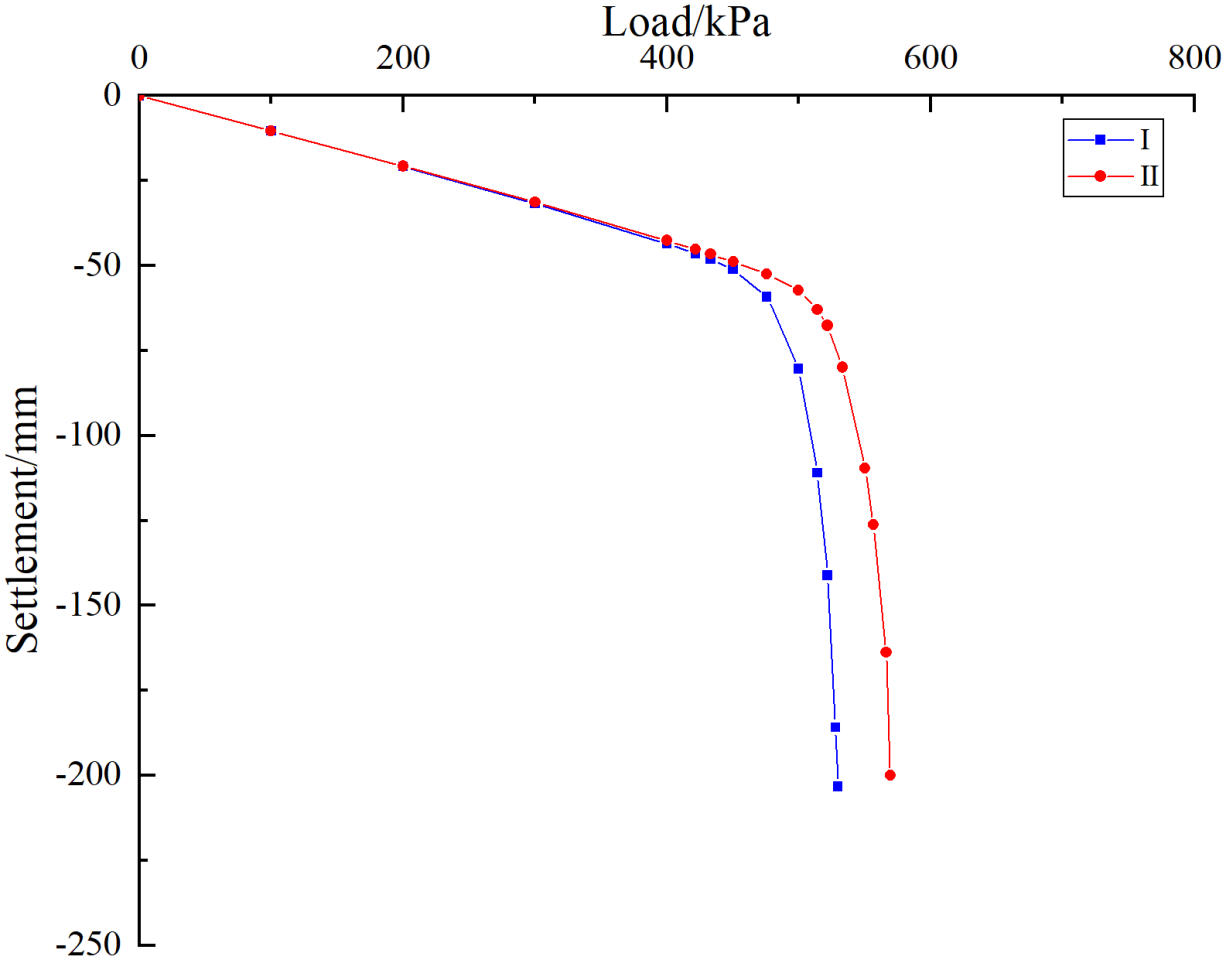
Load-settlement curve of pile top.

As shown in Fig 20, when the foundation is subjected to external load, both curves are in the linear stage at the initial loading stage. Although they show certain differences as the load increases, the difference is not large. The change of relative position of karst cave has little influence on the bearing capacity of pile group foundation. As the load continues to increase, the curve begins to enter the nonlinear stage, at which time the influence of the relative position of the cave on the bearing characteristics of the pile group foundation begins to appear. When the cave is located at the edge of the pile group (case I), the load-settlement curve enters the nonlinear stage quickly, and an obvious inflection point appears when the uniformly distributed load on the top of the cap is 425 kPa. When the cave is located in the middle area of the pile group (case II), the curve enters the nonlinear stage relatively slowly, and an obvious inflection point appears when the uniformly distributed load on the top of the cap is 465 kPa. According to the Technical Specifications for Building Foundation Pile Testing (JGJ 106–2014), when the load-settlement curve has an obvious steep drop section, the load at the starting point of the steep drop section is taken as the ultimate load. For the slowly varying curve, the settlement value corresponding to the ultimate bearing capacity is determined based on engineering experience and specific requirements. Generally, the load corresponding to the total settlement of the pile top reaches 40 mm is the ultimate load. It can be seen that the ultimate bearing capacity of the pile group foundation corresponding to cases Ⅰ and Ⅱ is 425 kPa and 465 kPa respectively. The comparison results show that the bearing capacity of the pile group foundation is better when there is a cave in the middle than when there is a cave in the edge, which reflects the influence of the pile group effect on the bearing characteristics of the pile foundation. The reason for this difference may be that the pile foundation in the “middle” part is subject to more constraints, and the cave has less impact on the pile foundation in the “middle” part of the pile group system.

**Fig 21:**
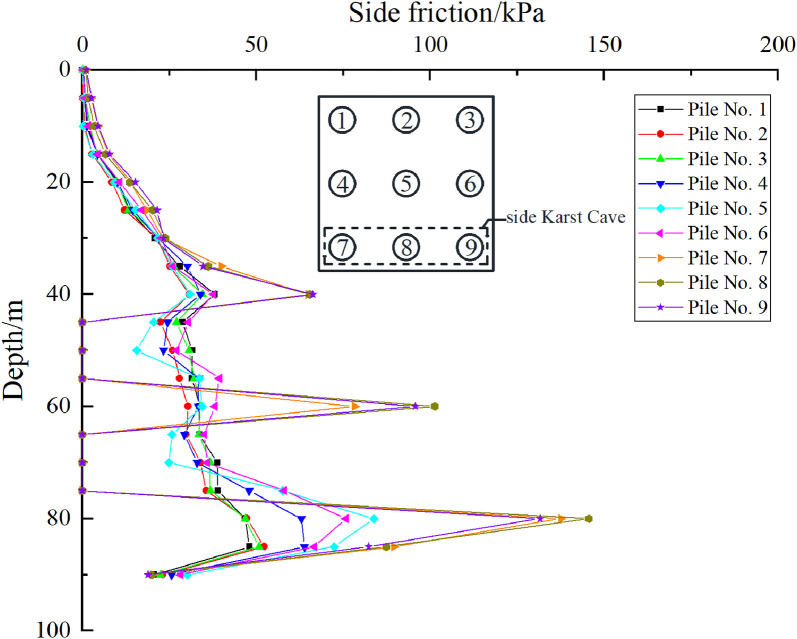
Side friction of each pile foundation of case I.

**Fig 22:**
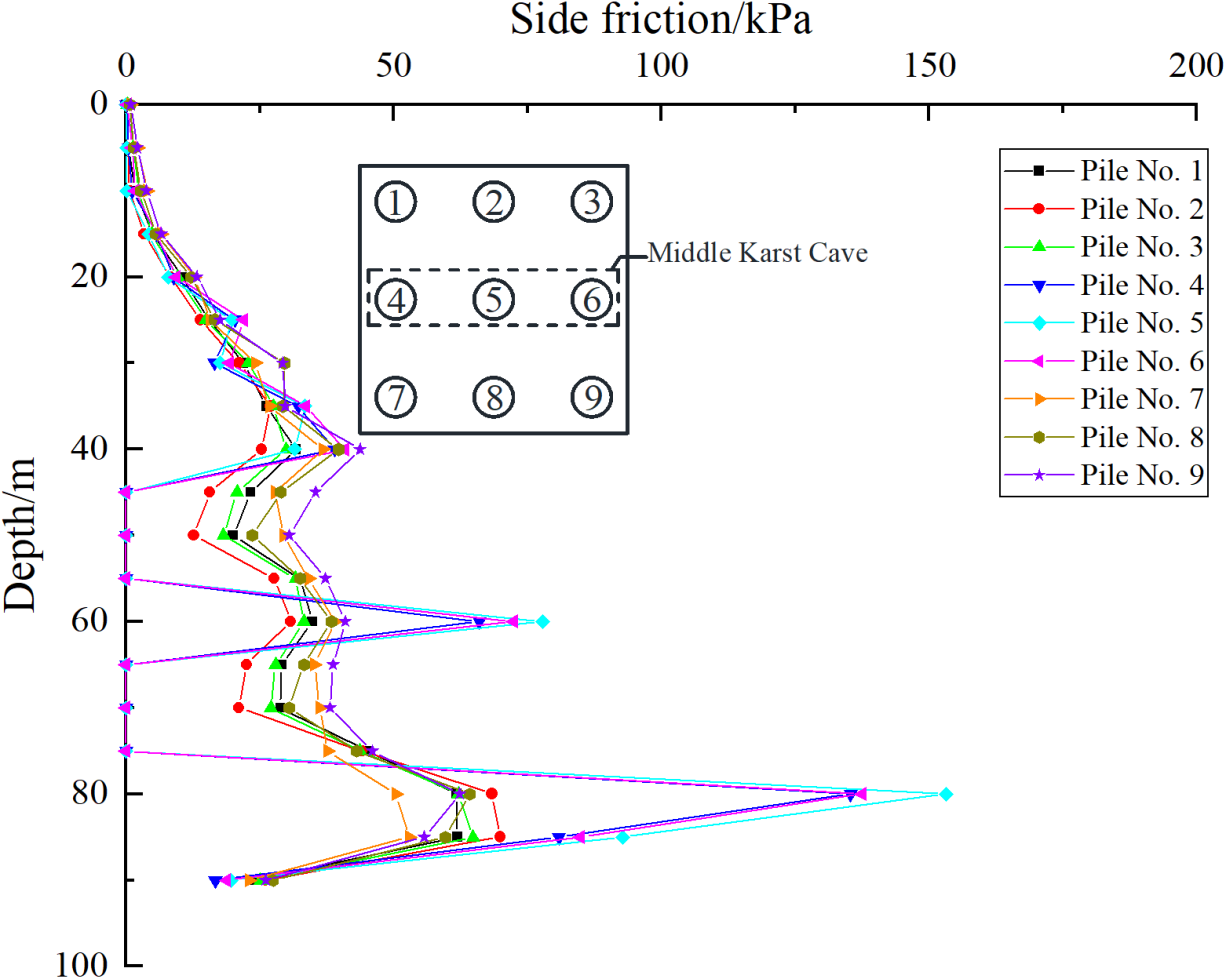
Side friction of each pile foundation of case Ⅱ.

Taking the uniformly distributed load of 300 kPa on the cap of pile group foundation as an example, the influence of beaded caves on the bearing capacity characteristics of pile foundation is studied. As shown in Figs 21, 22, for case I, pile foundations 1–3 are far away from the cave, and the cave has little impact on them. Therefore, the corresponding lateral friction resistance is almost the same as that of the pile group without the influence of the cave. However, pile foundations 4–6 are relatively close to the karst cave, resulting in the loss of some pile side friction resistance in the soil layer corresponding to the karst cave section. As shown in Fig 22, for case II, pile foundations 1–3 and 7–9 are all close to the cave. Therefore, each pile foundation loses part of the pile side friction in the corresponding soil layer of the cave section, but the overall impact is not obvious. When the karst cave is located at the edge or below the middle area of the pile group foundation, the bearing characteristics of the three piles passing through the karst cave are quite different from those of the ordinary pile group foundation. Specifically, the existence of the karst cave causes a direct loss of the pile side friction resistance of the pile foundation, and the bearing capacity changes.

**Fig 23:**
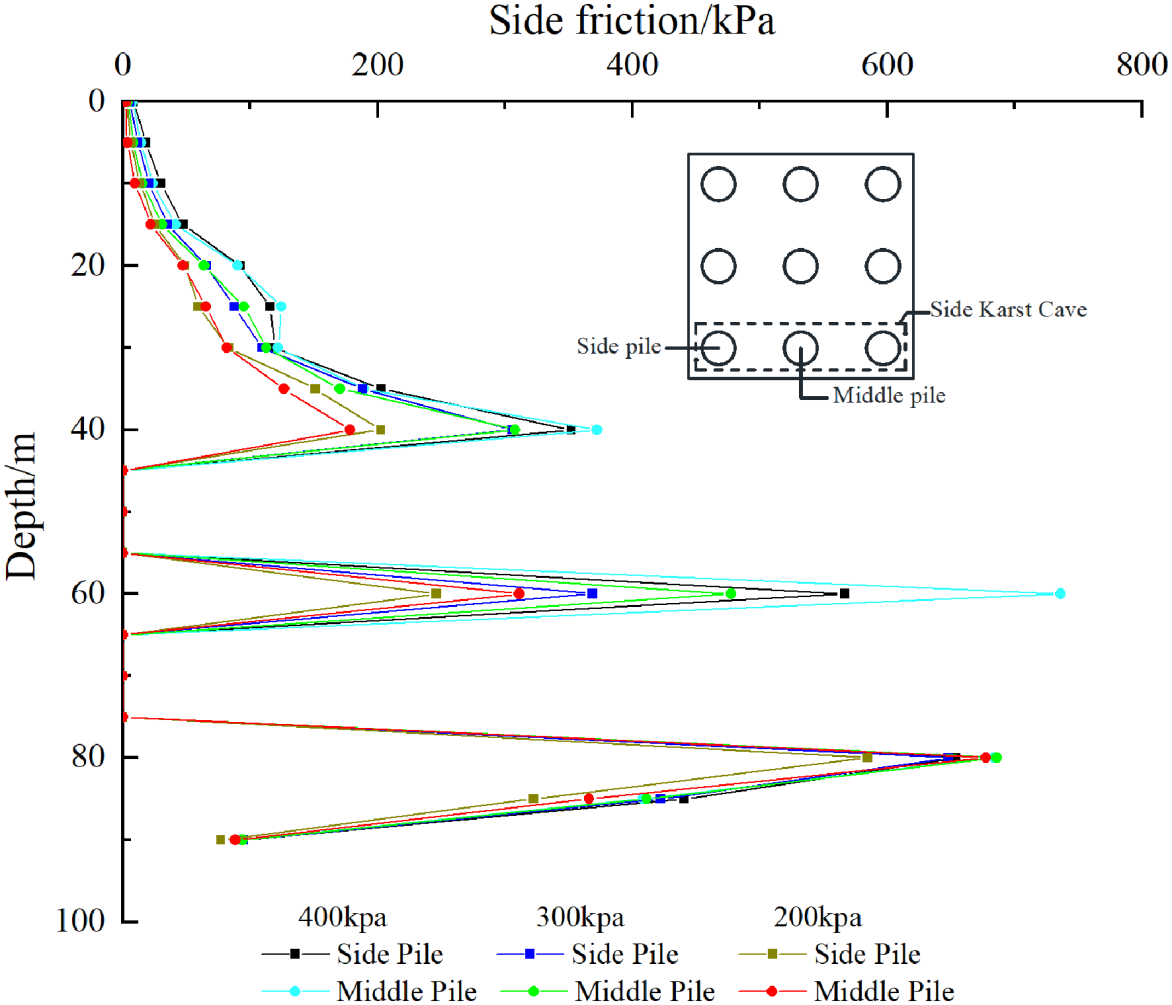
Side friction resistance of piles passing through karst caves of case Ⅰ.

**Fig 24:**
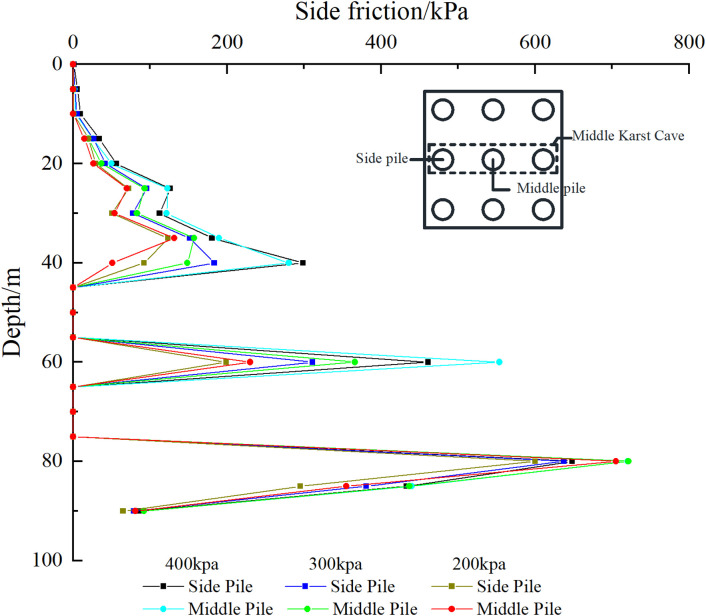
Side friction resistance of piles passing through karst caves of case Ⅱ.

The pile side friction resistance analysis of the three pile foundations passing through the cave was further conducted. For the convenience of description, the outer pile foundations of the three pile foundations passing through the cave are called “side piles” and the middle pile foundations are called “middle piles”. As shown in Figs 23, 24, the existence of karst caves makes the distribution of pile side friction significantly different from that of conventional pile foundations. Specifically, there are multiple maximum values of pile side friction along the depth direction, and the number of maximum values is affected by the number of karst caves that the pile foundation passes through. For the pile foundation passing through the beaded cave, the maximum value of the lateral friction resistance is mainly distributed in the overlying rock layer of the cave, the interlayer in the cave, and the rock layer below the cave. For the overlying rock strata, due to the constraint of the soil layer above the cave, the pile-soil interaction in this area is enhanced, resulting in the concentration of friction resistance on the pile side. For the interlayer, the stress condition is extremely complex, and the pile side friction resistance of the pile foundation at each position is distributed in an isosceles triangle. The maximum pile side friction resistance generally appears in the center of the interlayer. The bite effect of the pile-rock interface here is better than that of the free surface area above and below the interlayer close to the cave. The side friction resistance of the pile in the lower rock layer of the cave increases along the depth of the pile foundation and reaches a maximum value within a small thickness range. Its increasing rate is basically the same as that of the middle rock layer. After that, the side friction resistance gradually decreases with the increase of depth, and the load begins to be transferred to the pile end.

For case I, when the uniformly distributed load on the cap is 200–400 kPa, the first maximum points of the side piles and the middle piles appear at a depth of 40 m in the rock and soil layer. At this time, the side friction resistance of the side piles and the middle piles in the soil layer has been fully exerted.

For case II, when the uniformly distributed load on the cap is 200 kPa, the first maximum points of the side piles and the middle piles appear at the rock-soil interface (35 m), at which time the pile side friction resistance of the soil layer has not been fully exerted. When the uniformly distributed load on the cap is 300 kPa, the first maximum point of the side pile appears at a depth of 40 m in the rock and soil layer. At this time, the pile side friction resistance of the soil layer has been fully exerted, and the maximum pile side friction resistance is 190 kPa. After that, it changes little with the increase of the load on the cap. Under the same conditions, the maximum point of the middle pile is still at the rock-soil boundary (35 m). This is because the pile foundation in the “middle part” is subject to more constraints in the pile group system, and the load distribution is relatively more uniform. At the same time, due to the influence of the stress reduction of the pile group effect, the side friction resistance of the middle pile in the soil layer is not fully utilized. When the uniformly distributed load on the cap is 400 kPa, the side friction resistance of the side piles and corner piles in the soil layer is fully exerted, and the maximum value appears at a depth of 40 m in the rock and soil layer.

**Fig 25:**
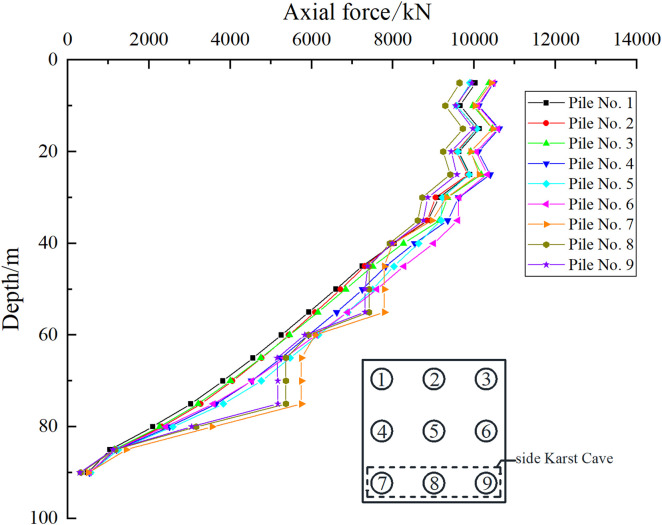
Axial force of each pile foundation of case Ⅰ.

**Fig 26:**
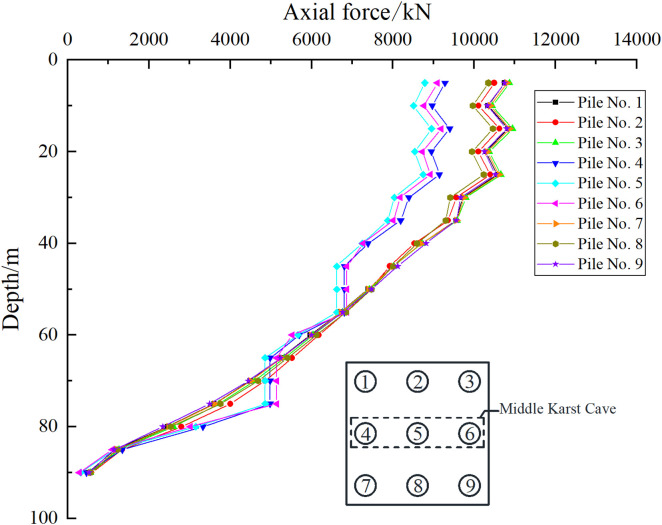
Axial force of each pile foundation of case Ⅱ.

As shown in Figs 25, 26, when the uniformly distributed load on the pile foundation cap is 300 kPa, for Case I, the axial force transmission mode of pile foundations 1–6 is consistent with that of the pile foundations without cave influence, and the axial force fluctuates in different soil layers. However, the existence of the cave changes the axial force transmission process of pile foundations 7–9, and the axial force variation trend is quite different from that of ordinary pile groups. For case II, the axial force transmission mode of pile foundations 1–3 and 7–9 is consistent with that of ordinary pile group foundations, while the performance of pile foundations 4–6 that pass through the cave is different. Therefore, we need to focus on analyzing the changing trend of the axial force of pile foundations crossing the cave area.

**Fig 27:**
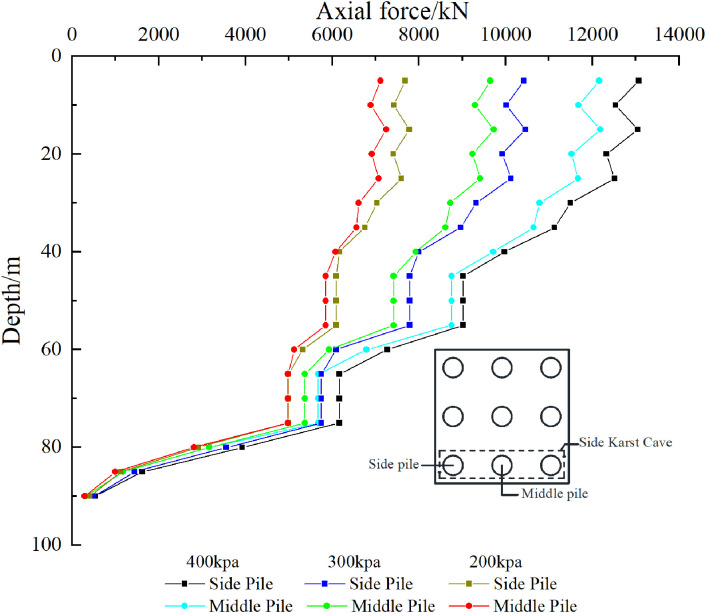
Axial force of pile foundation through karst cave of case Ⅰ.

**Fig 28:**
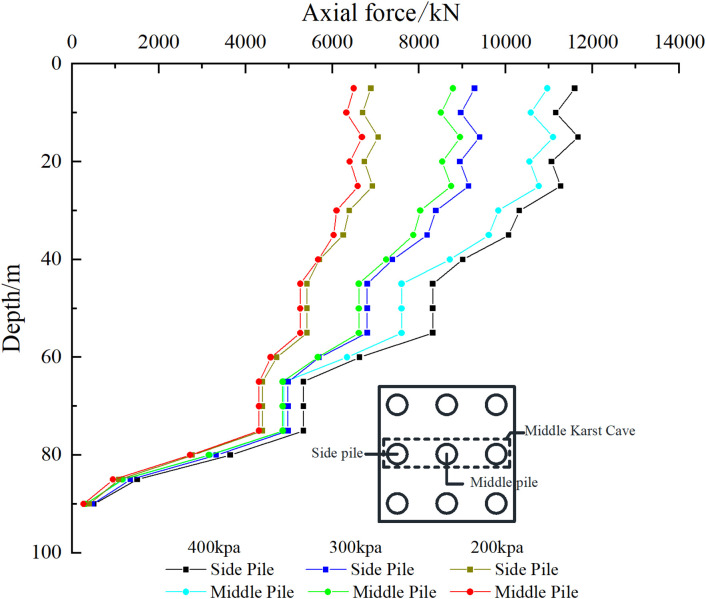
Axial force of pile foundation through karst cave of case Ⅱ.

As shown in Figs 27, 28, the presence of the cave causes the pile foundation to lose contact with the soil, resulting in an open surface. The transfer of load in the cave section is not affected by the soil, so the axial force of the pile foundation in this section remains unchanged. The axial force undergoes “tortuous” changes in the soil layer. The main reason is that the soil layer is composed of three parts: miscellaneous fill soil, silt soil and silty clay. Due to the differences in the physical and mechanical properties of the soil layer, the distribution of the friction resistance on the pile side is uneven, which affects the transmission of the axial force of the pile foundation. The minimum point of the pile axial force in the soil layer is mainly distributed at the junction of two layers of soil with different compositions, and the pile axial force in the rock layer decreases with the increase of the pile depth. Taking the pile depth of 35 m as an example, for case I, when the load on the cap is 200 kPa, the axial force of the side pile is 6686.35 kN and the axial force of the middle pile is 6495.42 kN. When the load on the cap is 300 kPa, the axial force of the side pile is 8827.63 kN and the axial force of the middle pile is 8481.28 kN. When the load on the cap is 400 kPa, the axial force of the side pile is 10907 kN and the axial force of the middle pile is 10431.24 kN. For case II, the load on the cap is 200 kPa, the axial force of the side pile is 6193.67 kN, and the axial force of the middle pile is 5974.51 kN. When the load on the cap is 300 kPa, the axial force of the side pile is 8069.66 kN, and the axial force of the middle pile is 7756.09 kN. When the load on the cap is 400 kPa, the axial force of the side pile is 9870.49 kN, and the axial force of the middle pile is 9420.46 kN. It can be found that as the external load increases, the difference in axial force between the side pile and the middle pile becomes larger and larger. When the load is small, the effect of the cave on the pile foundation is not obvious. As the load increases, the effect of the cave on the pile foundation becomes significant, and the proportion of the load shared by the outer pile foundation increases.

### 4.3. Verification of rationality of settlement calculation equation

**Fig 29:**
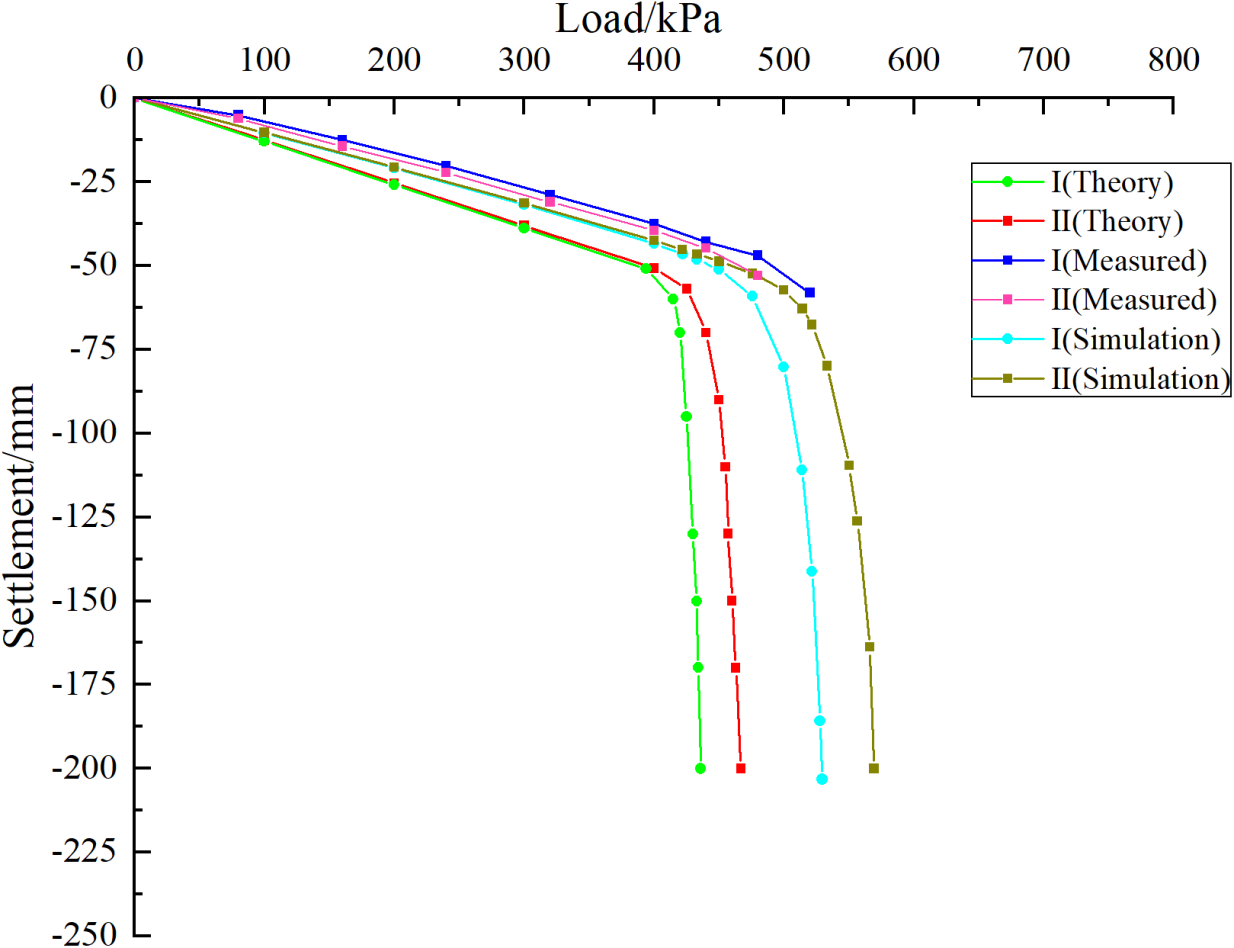
Comparison of pile foundation bearing characteristic curves.

Taking the pile foundation project in the karst area of the Science and Technology Manufacturing Park project in Bai-Yun District as an example, the rationality verification analysis of the settlement calculation equation of pile group foundation under the geological conditions of beaded caves proposed in this paper was carried out. A comparative study of equations (23–24) is conducted by combining theoretical analysis, field measurements and numerical simulation. As shown in Fig 29, the results of theoretical analysis, field monitoring and numerical simulation show that the curves are in a linear stage before the pile group foundation reaches its ultimate bearing capacity. In this stage, the influence of the cave on the bearing capacity of the pile foundation is small when it is located in different areas of the pile group. As the uniformly distributed load on the foundation increases, the influence of the relative position of the cave on the bearing characteristics of the pile foundation begins to emerge. Both theoretical derivation and simulation results show that when the cave is located in the middle area of the pile group foundation, the ultimate bearing capacity yield point of the pile foundation is larger. In addition, the measured data show that the settlement of the pile group foundation increases sharply after reaching the ultimate bearing capacity, which is basically consistent with the trend of theoretical derivation and simulation results. Taking the pile-soil mechanical parameters in Table 7 as the original data and bringing them into Equations (10), (15–17) and (23–24), it can be obtained that the corresponding ultimate bearing capacity when the cave is located in the middle area of the pile group foundation is 440.23 kPa. Similarly, the ultimate bearing capacity when the cave is located in the edge area of the pile group foundation is 395.45 kPa. Numerical simulation results show that the ultimate bearing capacities of the karst caves located in the middle and edge areas of the pile group foundation are 465 kPa and 425 kPa, respectively. Calculations indicate that when the cave is located at the edge and in the middle of the pile group foundation, the relative errors between numerical simulation and theoretical results are 7.47% and 5.63%, respectively. Field measurements show that when the karst cave is located at the centre of the pile group, the ultimate bearing capacity is approximately 480 kPa, which is about 9.03% lower than the theoretical prediction. When the cave is situated at the edge of the pile group, the ultimate bearing capacity is around 440 kPa, approximately 11.68% lower than predicted. The above error analysis results prove that the settlement calculation equation of pile group foundation introduced in this paper is reasonable to a certain extent. In this study, discrepancies were observed between theoretical, numerical, and field data. These arise because theoretical and numerical models often rely on assumptions and simplifications that may not fully reflect actual field conditions. Factors such as soil layer uniformity, pile-soil interactions, and idealized analytical models can all affect the results. In addition, differences between assumed input parameters and complex site-specific properties, such as soil elastic modulus and friction coefficient, also contribute to discrepancies between theory, simulation, and field data. In summary, the discrepancies result from multiple factors, including uncertainties in input parameters, simplifications in model assumptions, and measurement errors in the field. Future research should aim to minimise these discrepancies through more accurate field measurements and refined analytical models.

## 5. Discussion

### 5.1. Analysis of pile group bearing characteristics when a single pile penetrates a beaded cave

**Fig 30:**
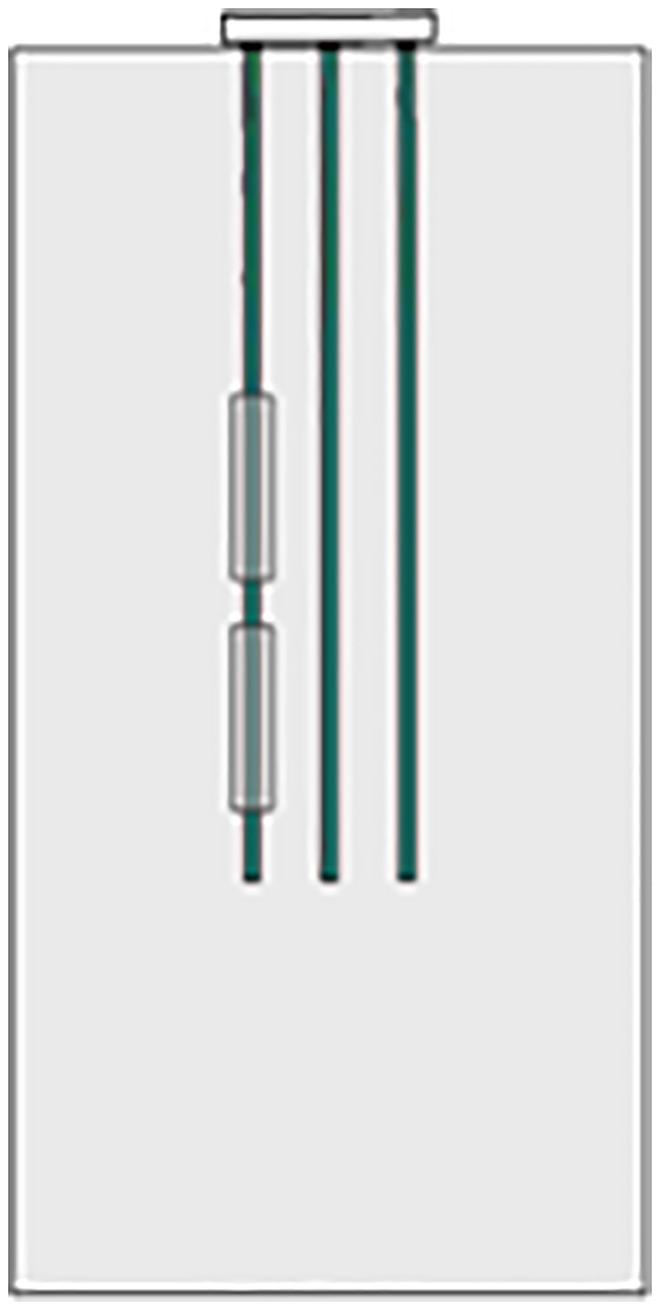
Cross-sectional diagram of cave location.

**Fig 31:**
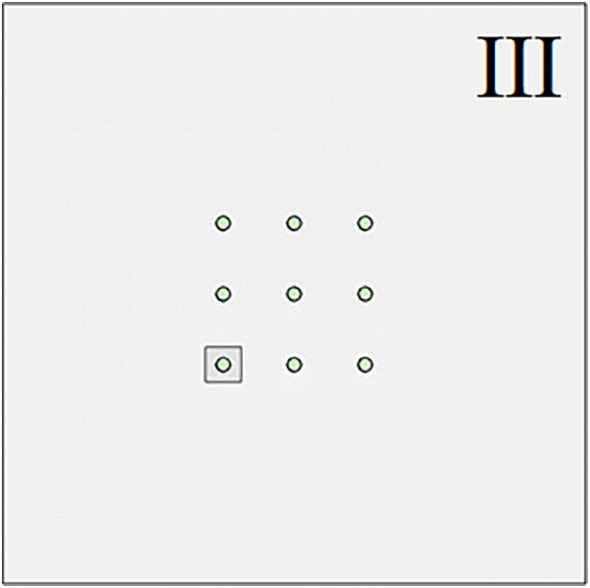
Case Ⅲ.

**Fig 32:**
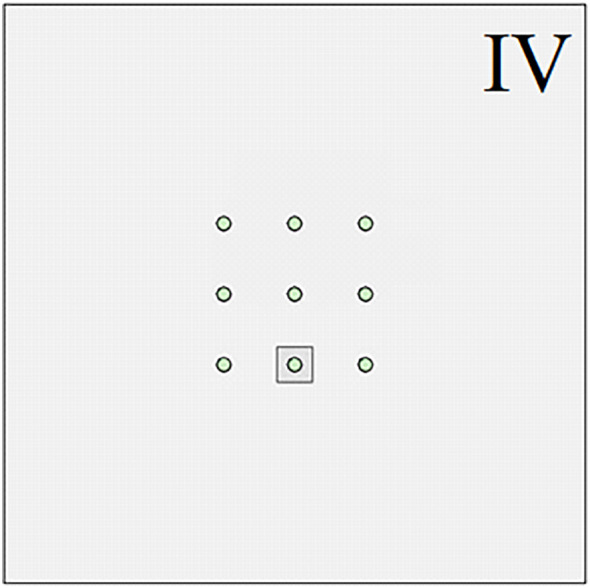
Case Ⅳ.

**Fig 33:**
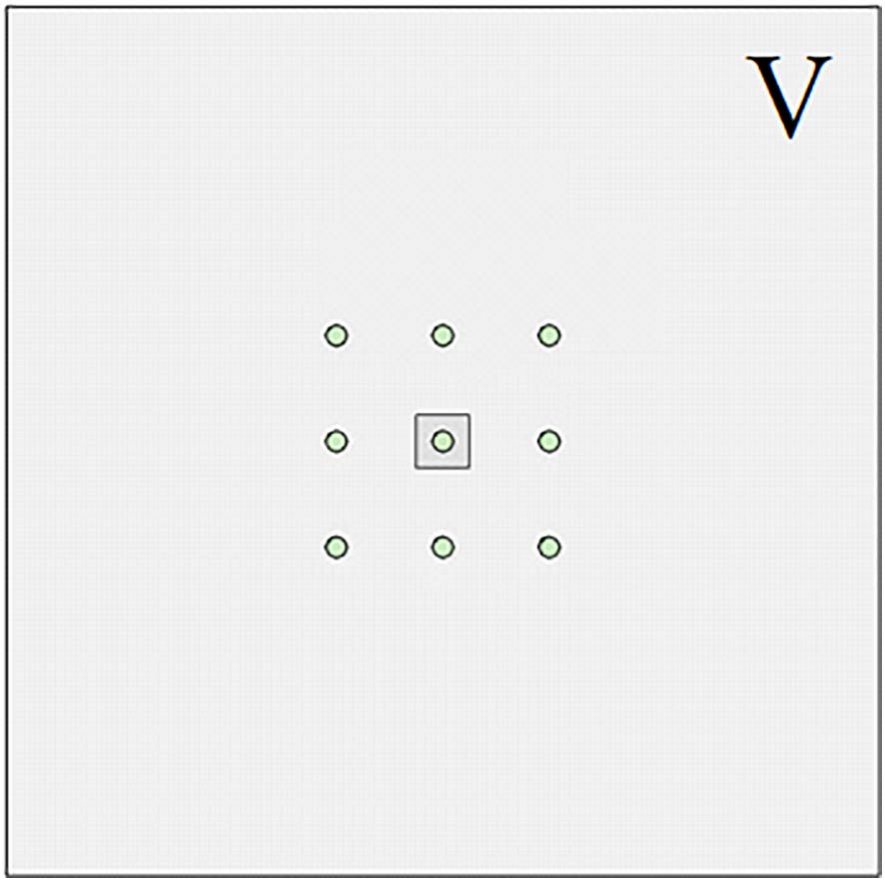
Case Ⅴ.

The above research is mainly aimed at the engineering problem of pile group foundation crossing large karst landforms. However, the size and shape of the karst caves contained in the strata are not exactly the same or similar. In actual engineering, there are often situations where only a single pile foundation in the pile group system is buried under or passes through a karst cave. In order to further study the influence of complex karst geological conditions on the bearing capacity of pile groups, numerical simulation was used to explore the evolution of the bearing characteristics of pile foundations when single piles at different pile group positions passed through a karst cave. As shown in Figs 30–33, when the size of the cave is 3.75 × 3.75 × 10 m, the cave is located directly below the corner piles, side piles, and middle piles of the entire pile group system. Similar to the above, the situation in Fig 31 is called III, the situation in Fig 32 is called IV, and the situation in Fig 33 is called V.

**Fig 34:**
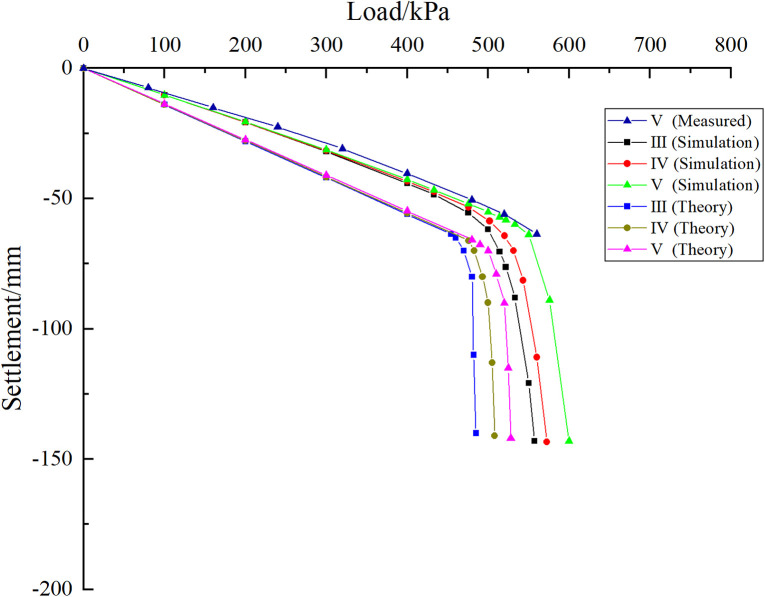
Load-settlement curves of corner, side and middle piles passing through caves.

As shown in Fig 34, at the initial stage of simulated loading, the three curves are all in the linear stage, and the influence of the cave on the pile group foundation is relatively small. As the external load increases, the curve begins to enter the nonlinear stage. At this time, the impact of different positions of the cave on the bearing characteristics of the pile foundation begins to appear. Among them, condition III enters the nonlinear stage earlier than condition IV and condition V. According to the judgment standard of the ultimate bearing capacity of pile group foundation, the ultimate bearing capacity of pile group foundation corresponding to III is 455 kPa, the ultimate bearing capacity of pile group foundation corresponding to IV is 472 kPa, and the ultimate bearing capacity of pile group foundation corresponding to V is about 505 kPa. Substituting the original mechanical parameters of the rock and soil into equation (18–20), the ultimate bearing capacity of the corresponding pile group foundation and its corresponding settlement yield point can be obtained. The theoretical calculation results show that the ultimate bearing capacity of the pile group foundation corresponding to III is 430.15 kPa, the ultimate bearing capacity of the pile group foundation corresponding to IV is 451.75 kPa, and the ultimate bearing capacity of the pile group foundation corresponding to V is 476.04 kPa. It can be found that when the cave is located directly below the corner pile area of the pile group foundation, the relative error of the numerical simulation result compared with the theoretical calculation result is 5.78%. Similarly, when the cave is located in the side pile area and the middle pile area of the pile group foundation, the relative errors are 4.48% and 6.08% respectively. Under monitoring condition V, the ultimate bearing capacity is approximately 520 kPa. The deviation between the measured results and the theoretical analysis is about 9.24%, which remains within an acceptable range and thereby substantiates the applicability of the theoretical method.

Both numerical simulation and theoretical analysis results show that the order of ultimate bearing capacity of pile foundations corresponding to the three working conditions is: V > IV > III. From the above results, it can be seen that the influence of karst caves on pile foundations with more original constraints is relatively small in pile group foundations.

### 5.2. Analysis of the coupling mechanism between cave location and pile foundation response

This study systematically examines the influence of cave location on the bearing capacity and settlement behavior of pile foundations. However, the settlement performance of pile group foundations is affected by cave location as well as by factors such as pile length, spacing, and number. Therefore, analyzing settlement behavior under different working conditions helps clarify the coupling mechanism between cave location and pile foundation response and provides a basis for optimizing pile foundation design.

The study systematically investigates how the location of karst caves affects the bearing capacity and settlement behavior of pile foundations with varying pile lengths, spacings, and numbers. Settlement behavior under different working conditions is compared to assess the influence of cave location on pile foundation performance and to clarify the coupling effects within pile foundation systems under complex conditions

#### 5.2.1. Influence of pile length on bearing characteristics of pile groups.

**Fig 35:**
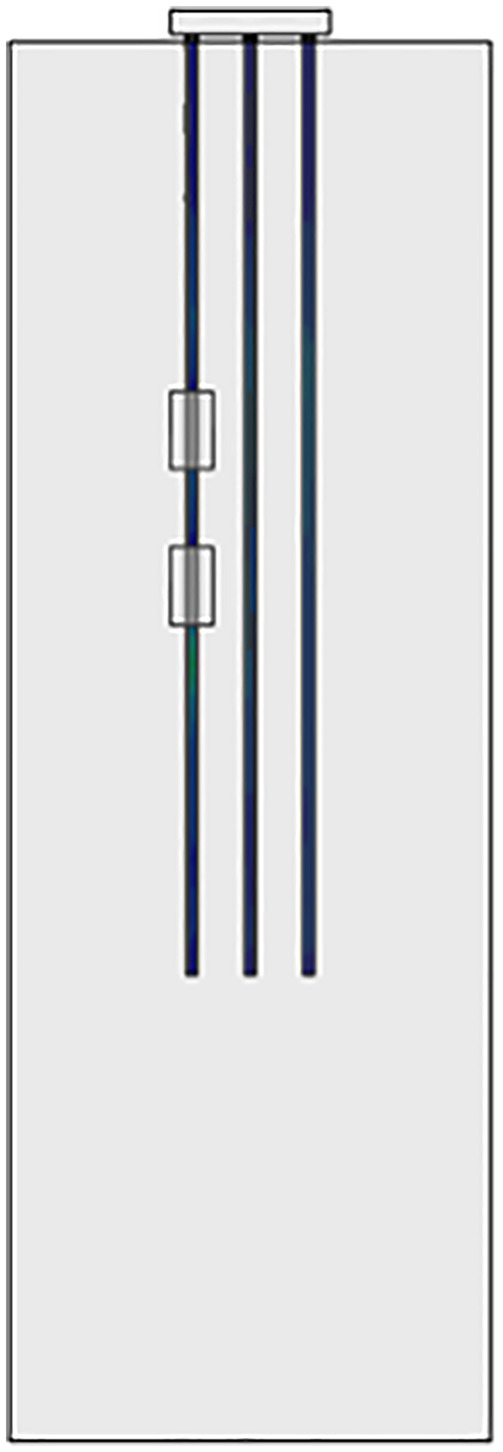
Pile length model of 121 m.

**Fig 36:**
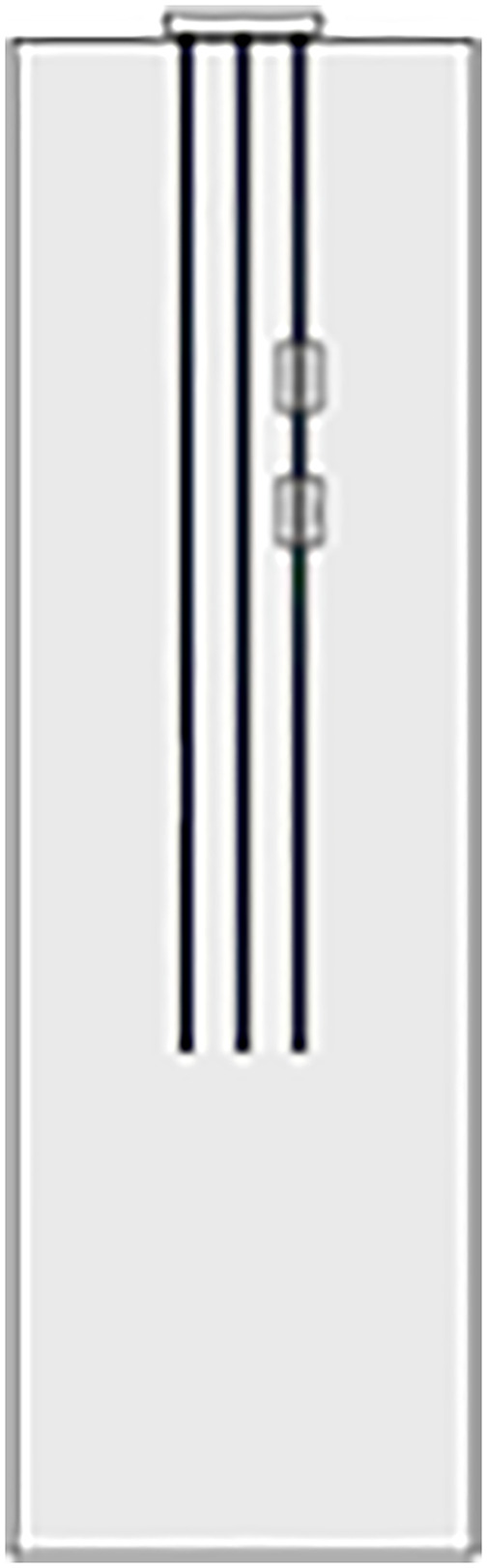
Pile length model of 151 m.

We established an analytical model with varying pile lengths to investigate the interaction between pile length and cave location. The additional models were established with pile lengths of 121 m and 151 m, maintaining a pile spacing of 5*D*. The model configuration is shown in Figs 35, 36.

**Fig 37:**
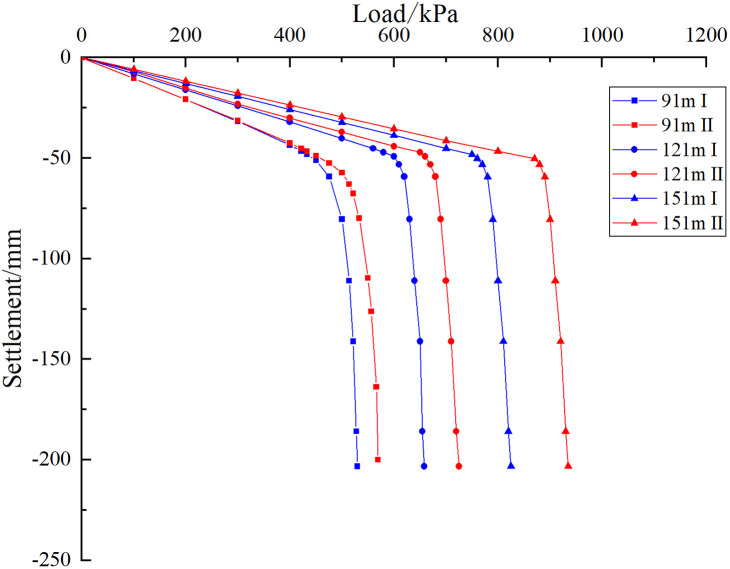
Load-settlement curves of models with different pile lengths.

As shown in Fig 37, the settlement behavior of the pile group foundations exhibits similar trends for all three pile lengths. This similarity results from the consistency of all model parameters and boundary conditions, except for pile length and overall model height. For a pile length of 91 m, the ultimate bearing capacity of the pile group foundation is 430 kPa when the cave is located at the edge. When the cave is situated at the centre, the ultimate bearing capacity increases to 475 kPa, representing a 10.46% rise compared with the edge cave condition. For a pile length of 121 m, the ultimate bearing capacity is 600 kPa when the cave is located at the edge. When the cave is situated at the centre, the ultimate bearing capacity rises to 680 kPa, representing an increase of 13.33% compared with the edge cave condition. For a pile length of 151 m, the ultimate bearing capacity is 760 kPa when the cave is located at the edge. When the cave is situated at the centre, the ultimate bearing capacity rises to 880 kPa, corresponding to a 15.79% increase compared with the edge cave condition. The results indicate that longer pile foundations exhibit a more pronounced pile group effect, higher reduction rates of the pile group effect, and a stronger influence of cave location on the settlement of the pile group foundation.

#### 5.2.2. Influence of pile spacing on bearing characteristics of pile groups.

**Fig 38:**
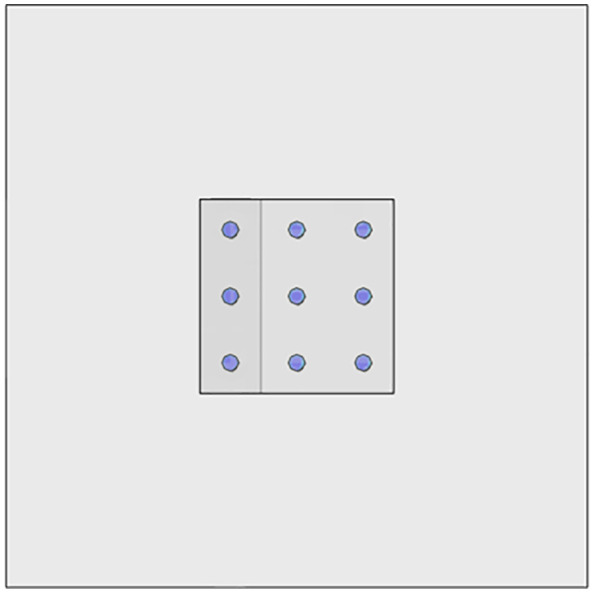
4*D* pile spacing model.

**Fig 39:**
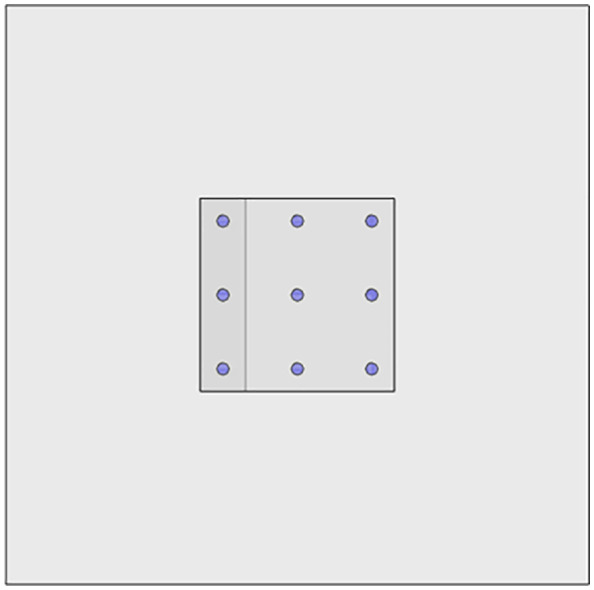
6*D* pile spacing model.

We established analytical models with varying pile spacings to investigate the interaction between pile spacing and cave location. The additional models were established with pile spacings of 4*D* and 6*D*, each with a pile length of 91 m. The model configuration is shown in Figs 38, 39.

**Fig 40:**
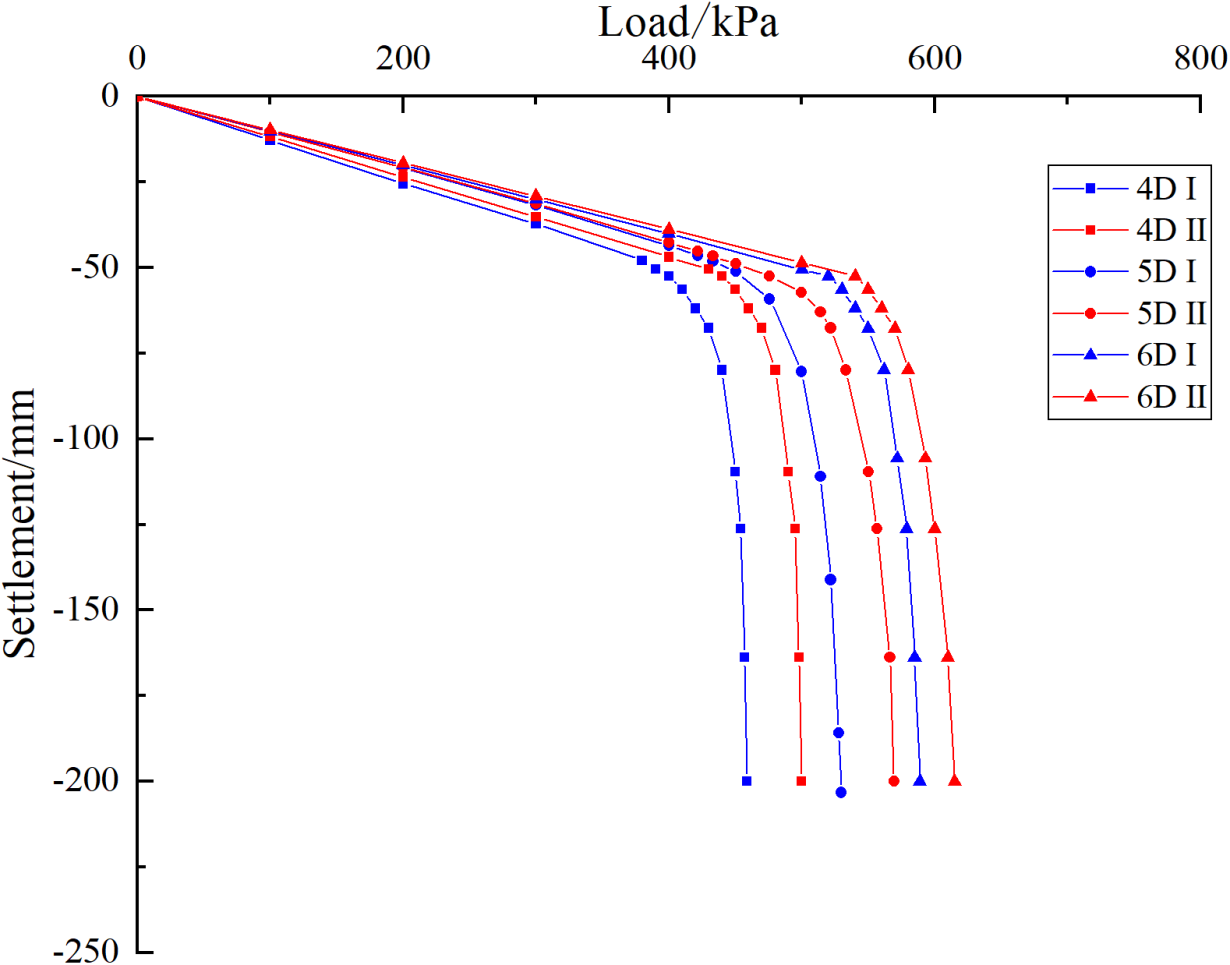
Load-settlement curves of models with different pile spacing.

As shown in Fig 40, for a pile spacing of 4*D*, the ultimate bearing capacity of the pile group foundation is 380 kPa when the cave is located at the edge. When the cave is situated at the centre, the ultimate bearing capacity increases to 425 kPa, representing an 11.84% rise compared with the edge cave condition. For a pile spacing of 5*D*, the ultimate bearing capacity is 430 kPa under the edge cave condition and 475 kPa under the central cave condition, corresponding to a 10.46% increase. For a pile spacing of 6*D*, the ultimate bearing capacities are 510 kPa and 550 kPa for the edge and central cave conditions, respectively, indicating a 7.84% increase. The results indicate that smaller pile spacing amplifies the pile group effect, increases the reduction rate, and strengthens the influence of cave location on the settlement behavior of the pile group foundation.

#### 5.2.3. Analysis of the influence of pile number on bearing characteristics of pile group foundation.

**Fig 41:**
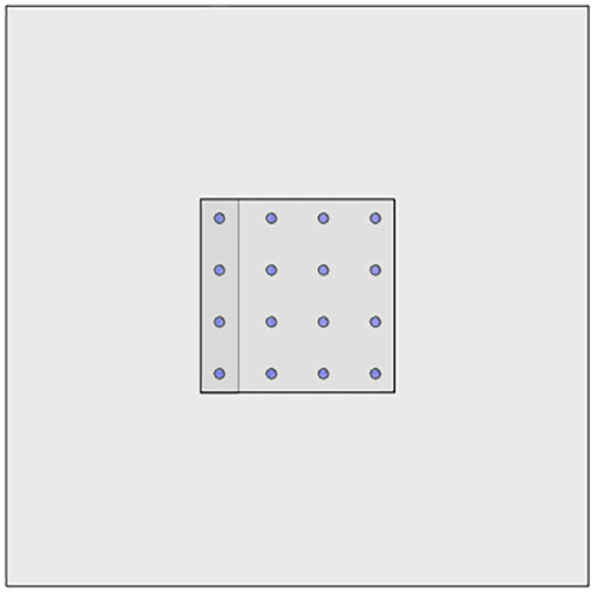
4 × 4pile group foundation model.

**Fig 42:**
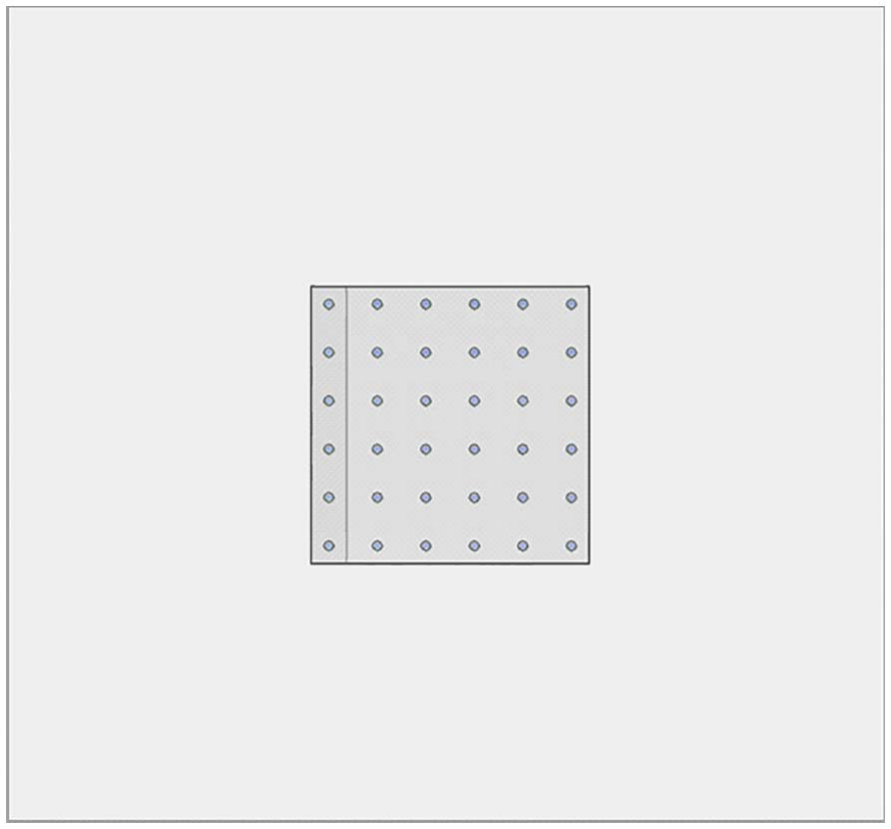
6 × 6 pile group foundation model.

We established analytical models with varying pile numbers to investigate the interaction between pile number and cave location. The additional models were established as 4 × 4 and 6 × 6 pile group foundations. The model configuration is shown in Fig 41–42.

**Fig 43:**
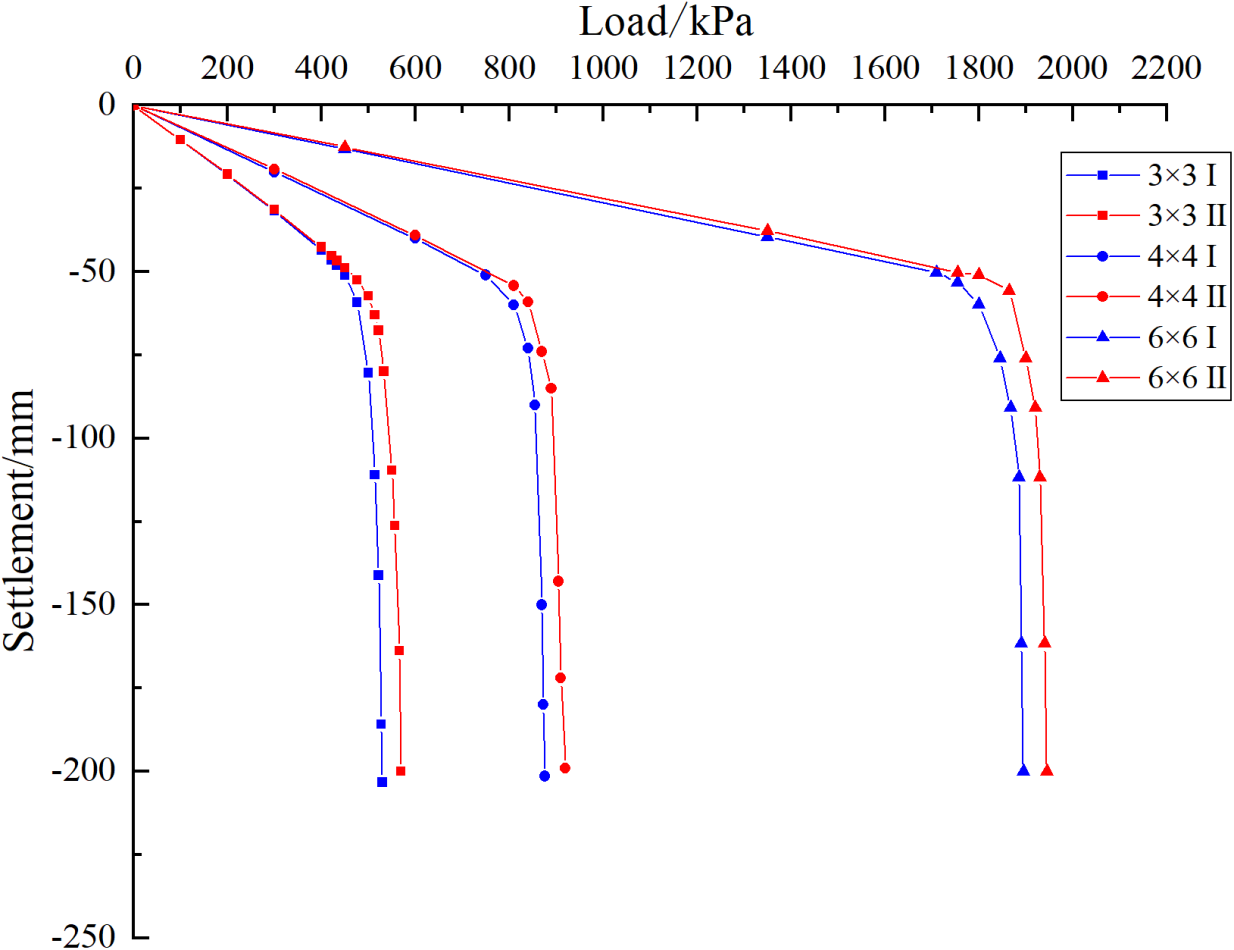
Load-settlement curves of models with different numbers of piles.

As shown in Fig 43, the ultimate bearing capacity of the 3 × 3 pile group foundation is 430 kPa when the cave is located at the edge. When the cave is centrally located, the bearing capacity increases to 475 kPa, representing a 10.46% improvement over the edge cave condition. For the 4 × 4 pile group foundation, the ultimate bearing capacity rises from 750 kPa at the edge to 830 kPa with a central cave, a 10.67% increase. Similarly, for the 6 × 6 pile group foundation, the ultimate bearing capacity increases from 1700 kPa at the edge to 1860 kPa with a central cave, reflecting a 9.41% increase. These results indicate that the number of piles has a limited effect on pile group behavior. When pile spacing is at least 3D, each pile is mainly influenced by its eight immediate neighbours, with minimal impact from more distant piles. Consequently, the settlement differences caused by cave location in the 6 × 6 and 4 × 4 pile groups closely resemble those observed in the 3 × 3 group. Furthermore, the reduction rate formula is independent of pile number and is influenced solely by pile length and spacing.

### 5.3. Comparison of settlement calculation methods

At present, the settlement calculation methods for pile group foundations in karst areas mainly include the traditional empirical method, the equivalent pile raft method, the Mindlin solution superposition method, the pile-soil stiffness ratio method and the (*T-Z*) load transfer method. In order to verify the superiority of the settlement calculation formula for pile group foundations proposed in this paper, we carried out relevant comparative calculations. The relevant theoretical model and calculation results are shown in Table 10. It is worth noting that this settlement calculation takes working condition AZK12 as an example, and the external load is assumed to be a uniformly distributed load of 400kPa.

**Table 10:** Calculation method for pile group settlement.

Calculation method	Calculation formula	parameter	Calculate the result
Traditional empirical method	$N=\frac{Q}{S_{e}E_{e}}$	*N* represents the settlement of the pile group foundation. *Q* denotes the total load. *S*_*e*_ is the equivalent pile foundation area. *E*_*e*_ is the equivalent deformation modulus, which reflects the combined behavior of the pile and soil.	53.9 mm
Equivalent pile raft method	$N=\frac{Q\cdot R_{g}}{n\cdot S_{P}e_{P}}+S_{S}$	*Q* is the total load of the pile group. *n* is the number of piles. *S*_*p*_ represents the cross-sectional area of a single pile. *E*_*p*_ denotes the elastic modulus of the pile. *R*g is the correction factor accounting for the pile group effect. *S*_*s*_ is the compression settlement of the foundation soil between piles.	35.94 mm
Mindlin solution superposition method	$N=\sum_{i=1}^{n}\frac{P_{i}}{4\pi G(1-v)}\left(\frac{\text{1}}{r_{i}}\right)$	*n* is the number of piles. *P*_*i*_ is the load on the *i*-th pile. *G* represents the shear modulus of the foundation soil. *ν* is Poisson’s ratio. *r*_*i*_ denotes the pile spacing or the distance from the load application point to the calculation point.	49.37 mm
Pile-soil stiffness ratio method	$N=\frac{E_{p}\cdot I_{p}}{E_{s}\cdot L^{4}}\cdot L$	*E*_*p*_ is the elastic modulus of the pile. *E*_*s*_ is the elastic modulus of the soil, and a weighted average is used for soils composed of multiple layers. *I*_*p*_ is the moment of inertia of the pile. *L* is the length of the pile.	50.48 mm
(*T*-*Z*) load transfer method	$N=\sum \frac{\Delta T(z)}{E_{p}A_{p}}\cdot \Delta z$	Δ *T*(*Z*) represents the incremental change in frictional resistance at the *Z*-th layer. *E*_*p*_ is the elastic modulus of the pile. *A*_*p*_ is the cross-sectional area of the pile. Δ *Z* denotes the height of the *Z*-th layer (i.e., the length of the discrete pile segment).	48.54 mm

Comparing the above calculation results with the actual monitoring results, the relative errors of the traditional empirical method, pile-soil stiffness ratio method and equivalent pile raft method are 23.79%, 15.94% and 17.46% respectively. The errors are too large and their applicability is limited. The errors of the calculation results of Mindlin solution superposition method and (T-Z) load transfer method compared with monitoring are within the controllable range, which are 13.39% and 11.48% respectively, but their iterative solution processes are too complicated. Compared with other calculation methods, the settlement value of the theoretical calculation method proposed in this paper is 47.46 mm, and the error compared with the actual monitoring is only 9%, which is more suitable for the calculation of pile group settlement in karst areas.

## 6. Conclusion

Relying on the pile foundation project of Bai-Yun District Science and Technology Manufacturing Park, this paper studies the bearing characteristics of pile group foundations in karst areas through theoretical analysis, field measurement and numerical simulation. The influence of the relative position of the beaded caves on the bearing capacity of the pile group foundation was analyzed in detail. The following conclusions were drawn:

a) Based on the theory of pile side friction loss in karst areas, the analytical equation for calculating the ultimate bearing capacity of pile groups is derived, and the bearing characteristic curve of the pile group partially crossing the karst landform is obtained through the layered comprehensive method. Relying on the pile foundation project of Bai-Yun District Science and Technology Manufacturing Park, the rationality of the theoretical model was verified through on-site monitoring and numerical simulation results. The verification results show that the calculation results of the theoretical model are in good agreement with the field measured data and numerical simulation results. The theoretical model can accurately reflect the bearing characteristics of pile group foundations when the caves are located in different areas.
b) The research results show that the influence of karst caves on the settlement behavior of pile group foundations is small in the linear stage, but significant in the nonlinear stage. When the caves are located in different areas below the pile group foundation, the ultimate bearing capacity of the pile group foundation and the corresponding settlement yield point are different. For the case of multiple piles crossing in a pile group foundation, the order of the ultimate bearing capacity of the pile group foundation is Ⅱ > Ⅰ. In practical pile foundation construction, adjusting the layout to avoid placing pile groups directly above karst caves can effectively enhance the overall bearing capacity of the foundation system. If avoiding caves is not feasible, the design may be adjusted so that the central pile intersects the cave, thereby enhancing the ultimate bearing capacity of the pile group. For the case of a single pile crossing in a pile group foundation, the order of the ultimate bearing capacity of the pile group foundation is Ⅴ > Ⅳ > Ⅲ. If a pile group foundation cannot avoid a cave, the design should be optimised to locate the central pile within the cave, thereby reducing its influence on the overall performance of the foundation.
c) The interplay among pile length, pile spacing, and the location of karst caves has a significant effect on the settlement behavior of pile group foundations. In contrast, the interaction between the number of piles and cave location has a minimal effect. The research results show that longer piles and reduced pile spacing amplify settlement variations related to cave location, whereas the number of piles has little effect on these variations.

## 7. Prospect

Based on the foundation project of Bai-Yun District Science and Technology Manufacturing Park in Guangzhou, this paper conducted relevant research on the bearing capacity and settlement calculation of pile groups under bead like karst geological conditions, and obtained relevant research results for this specific project. To better understand the influence of cave location on the bearing characteristics of pile group foundations, this study reviewed relevant literature [44,45] and moderately simplified the analysis model. The karst cavity is represented as a regular rectangular shape. The rock layer is considered uniform and intact, free of complex features such as cracks or joints. The surrounding rock and soil are treated as a continuous and homogeneous medium. However, these simplifications entail inherent limitations, leading to discrepancies between theoretical analyses, simulation results, and field measurements.

In the current research and analysis of pile foundation bearing capacity and settlement calculation in karst areas, the shape of the karst cave is usually assumed to be circular or rectangular, which can make the calculation more convenient. Studies [46,47] have shown that rectangular caves can better reflect the impact of caves on the bearing characteristics of pile foundations than circular caves. Therefore, we used rectangular caves to carry out numerical calculations in this paper, which can help the calculation convergence of the ABAQUS model. However, caves are usually irregular in shape, and rectangular caves cannot fully reflect the mechanical behavior and deformation characteristics of real caves in actual scenarios. Therefore, we will further restore the shape of real caves to improve the accuracy of simulation in subsequent research. Soil heterogeneity [48] and solidification conditions [49] also affect the bearing characteristics of pile foundations. Studies [50,51] show that soil heterogeneity induces uneven stress distribution in pile groups, resulting in differential settlement, greater variability in internal forces and deformations, abnormal bending moment and shear force patterns, and, in severe cases, structural damage to the piles. Moreover, soil solidification leads to considerable variations in strength and compressibility around the piles. To simplify the analysis and emphasise the influence of cave location on the bearing characteristics of pile groups, the numerical simulations assumed homogeneous soil and idealised solidification conditions. In subsequent research, we will integrate heterogeneous parameters with stochastic finite element methods to enhance model accuracy and applicability. The interaction between piles and soil is not a simple two-dimensional mechanical problem, but rather involves complex three-dimensional spatial effects [52–54]. The stress distribution in the soil surrounding the pile exhibits three-dimensional variations. The interaction between piles and soil occurs in the horizontal, vertical, and depth directions. In subsequent research, we will focus on analyzing the effects of three-dimensional pile-soil interaction on the bearing characteristics of pile group foundations.

## 8. Methods

The data used in this study were obtained from static load tests on pile foundations, carried out by the research team at the Bai-Yun District Science and Technology Manufacturing Park in Guangzhou. These tests formed part of the routine construction process and were authorized by the project owner for scientific research purposes. The study did not involve experiments in protected areas, humans, or animals and therefore required no additional permits.

## Supporting information

S1 Data Minimal dataset including raw values underlying all figures and tables. (ZIP)

## Abbreviations

*W*_*U*_: Ultimate bearing capacity of pile groups
*P*_*U*_: Ultimate bearing capacity of a single pile
P’ U: Actual bearing capacity of a single pile in a pile group system
*Q*_*S*_: Pile side friction
Q’ S: Side friction of a single pile in a pile group foundation
Q” S: Side friction of a single pile in a pile group foundation passing through a cave
*Q*_*S total*_: Total pile lateral friction of pile group foundation under the condition of crossing karst cave
D: Pile foundation diameter.
